# Conditional MaxRS Query for Evolving Spatial Data

**DOI:** 10.3389/fdata.2020.00020

**Published:** 2020-06-19

**Authors:** Muhammed Mas-ud Hussain, Mir Imtiaz Mostafiz, S. M. Farabi Mahmud, Goce Trajcevski, Mohammed Eunus Ali

**Affiliations:** ^1^Department of Electrical Engineering and Computer Science, Northwestern University, Evanston, IL, United States; ^2^Department of Computer Science and Engineering, Bangladesh University of Engineering and Technology, Dhaka, Bangladesh; ^3^Department of Electrical and Computer Engineering, Iowa State University, Ames, IA, United States

**Keywords:** maximizing range sum query, constrained query processing, conditional MaxRS, C-MaxRS, bulk data updates, bursty streams, spatial data streams, spatial indexing

## Abstract

We address the problem of maintaining the correct answer-sets to a novel query—*Conditional* Maximizing Range-Sum (C-MaxRS)—for spatial data. Given a set of 2D point objects, possibly with associated weights, the traditional MaxRS problem determines an optimal placement for an axes-parallel rectangle *r* so that the number—or, the weighted sum—of the objects in its interior is maximized. The peculiarities of C-MaxRS is that in many practical settings, the objects from a particular set—e.g., restaurants—can be of different types—e.g., fast-food, Asian, etc. The C-MaxRS problem deals with maximizing the overall sum—however, it also incorporates class-based constraints, i.e., placement of *r* such that a lower bound on the count/weighted-sum of objects of interests from particular classes is ensured. We first propose an efficient algorithm to handle the static C-MaxRS query and then extend the solution to handle dynamic settings, where new data may be inserted or some of the existing data deleted. Subsequently we focus on the specific case of bulk-updates, which is common in many applications—i.e., multiple data points being simultaneously inserted or deleted. We show that dealing with events one by one is not efficient when processing bulk updates and present a novel technique to cater to such scenarios, by creating an index over the bursty data on-the-fly and processing the collection of events in an aggregate manner. Our experiments over datasets of up to 100,000 objects show that the proposed solutions provide significant efficiency benefits over the naïve approaches.

## 1. Introduction

Rapid advances in accuracy and miniaturization of location-aware devices, such as GPS-enabled smartphones, and increased use of social networks services (e.g., check-in updates) have enabled a generation of large volumes of spatial data(e.g., Manyika et al., 2011). In addition to the *(location, time)* values, that data is often associated with other contextual attributes. Numerous methods for effective processing of various queries of interest in such settings—e.g., range, (*k*) nearest neighbor, reverse nearest-neighbor, skyline, etc.—have been proposed in the literature(cf., Zhang et al., 2003; Zhou et al., 2011; Issa and Damiani, 2016).

One particular spatial query that has received recent attention is the, so called, *Maximizing Range-Sum (MaxRS)* (Choi et al., 2014), which can be specified as follows: given a set of weighted spatial-point objects *O* and a rectangle *r* with fixed dimensions (i.e., *a* × *b*), MaxRS retrieves a location of *r* that maximizes the sum of the weights of the objects in its interior. Due to diverse applications of interest, variants of MaxRS (e.g., Phan et al., 2014; Amagata and Hara, 2016; Feng et al., 2016; Hussain et al., 2017a,b; Wongse-ammat et al., 2017; Liu et al., 2019, etc.) have been recently addressed by the spatial database and sensor network communities.

What motivates this work is the observation that in many practical scenarios, the members of the given set *O* of objects can be of different types, e.g., if *O* is a set of restaurants, then a given *o*_*i*_ ∈ *O* can belong to a different class from among fast-food, Asian, French, etc. Similarly, a vehicle can be a car, a truck, a motorcycle, and so on. In the settings where data can be classified in different (sub)categories, there might be class-based participation constraints when querying for the optimum region—i.e., a desired/minimum number of objects from particular classes inside *r*. However, due to updates in spatial databases—i.e., objects appearing and disappearing at different times—one needs to accommodate such dynamics too. Following two examples illustrate the problem:

*Example 1:* Consider a campaign scenario where a mobile headquarters has limited amount of staff and needs to be positioned for a period of time in a particular area. The US Census Bureau has multiple surveys on geographic distributions of income categories^1^ and, for effective outreach purposes, the campaign managers would like to ensure that within the limited reachability from the headquarters, the staff has covered a maximum amount of voters—with the constraint that a minimum amount of representative from different categories are included. This would correspond to the following query:

**Q1**: “*What should be the position of the headquarters at time* *t* *so that at least* κ_*i*_ *residents from each income Category*_*i*_ *can be reached, while maximizing the number of voters reached, during that campaign date.”*

*Example 2:* Consider the scenario of X's **Loon Project**^2^, where there are different types of users—premium (class A), regular (class B), and free (class C), and users can disconnect or reconnect anytime. In this context, consider the following query:

**Q2**: “*What should be the position of an Internet-providing balloon at time* *t* *to ensure that there are at least* Θ_*i*_ *users from each Class*_*i*_ *inside the balloon-coverage and the number of users in its coverage is maximized?”*.

It is not hard to adapt **Q1** and **Q2** to many other applications settings:—environmental tracking (e.g., optimizing a range-bounded continuous monitoring of different herds of animals with both highest density and diversity inside the region);—traffic monitoring (e.g., detecting ranges with densest trucks);—video-games (e.g., determining a position of maximal coverage in dynamic scenarios involving change of locations of players and different constraints).

We call such queries *Conditional Maximizing Range-Sum* (C-MaxRS) queries, a variant of the traditional MaxRS problem. For dynamic settings, where the objects can be inserted and/or deleted, we have *Conditional Maximizing Range-Sum with Data Updates* (C-MaxRS-DU) query.

**Figure 1:**
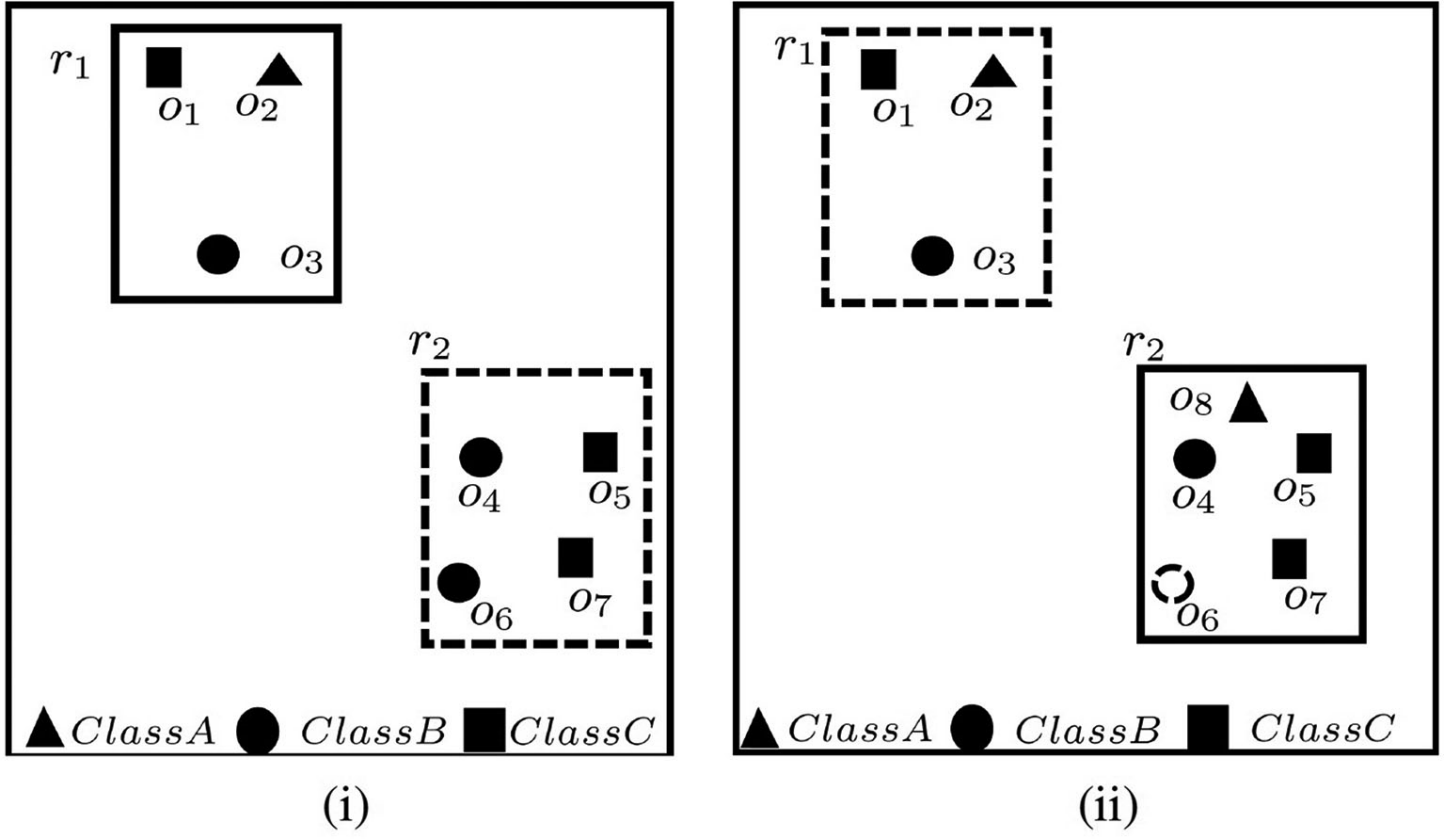
An example of C-MaxRS problem in spatial data updates at time **(i)** *t*_1_ and **(ii)** *t*_2_.

An illustration for C-MaxRS query in a setting of 7 users grouped into 3 classes (i.e., A, B, and C), and with a query rectangle size *a* × *b* (i.e., height *a* and width *b*) is shown in Figure 1. Assume that the participation constraint is that *the positioning of* *r* *must be such that at least 1 user is included from each of the classes A, B, and C, respectively*. There are two rectangles *r*_1_ and *r*_2_, with dimension *a* × *b*, that are candidates for the solution. However, upon closer inspection it turns out that although *r*_2_ contains most users (corresponding to the traditional MaxRS solution), it is *r*_1_ that is the sought-for solution for the C-MaxRS problem. Namely, *r*_2_ does not satisfy the participation constraints (see Figure 1i).

Now, suppose that at time *t*_2_, user *o*_6_ disconnects and a new user *o*_8_ joins the system. Then the C-MaxRS solution will need to be changed to *r*_2_ from *r*_1_ (see Figure 1ii).

Our key idea for efficient C-MaxRS processing is to partition the space and apply effective pruning rules for each partition to quickly update the result(s). The basic processing scheme follows the technique of spatial subdivision from Feng et al. (2016), dividing the space into a certain number of slices, whose local maximum points construct the candidate solution point set. In each slice, the subspace was divided into slabs which helps in reducing the solution space. To handle dynamic data stream scenarios, i.e., appearances and disappearances of objects, we propose two algorithms, *C-MaxRS*^+^ and *C-MaxRS*^−^, respectively, which works as a backbone for solving the constrained maximum range sum queries in the dynamic insertions/deletions settings (C-MaxRS-DU). Our novelty is in incorporating heuristics to reduce redundant calculations for the newly appeared or disappeared points, relying on two trees: a quadtree and a balanced binary search tree. Experiments over a wide range of parameters show that our approach outperforms the baseline algorithm by a factor of three to four, for both Gaussian and Uniform distribution of datasets.

The above idea for the C-MaxRS-DU algorithm takes an event-based approach, in the sense that C-MaxRS is evaluated (maintained) every time an event occurs, i.e., new point appears (*e*^+^) or an old point disappears (*e*^−^). This approach works efficiently when events are distributed fairly uniformly in the temporal domain and occur at different time instants that are enough apart for reevaluation to complete. However, the recent technological advancements and the availability of hand-held devices have enabled a large increase (or decrease) of the number of active/mobile users in multitude of location-aware applications in relatively short time-spans. In the context of Examples 1 and 2, this would correspond to the following scenarios:

*Example 1*: If the area involves businesses, then one would want to exploit the fact that many individuals may: (a) come (or leave) their place of work in the morning (or evening); (b) enter (or leave) restaurants during lunch-time; etc.

*Example 2*: In the settings of X's Loon Project, there can be multiple users disconnecting from the service simultaneously (within a short time span), or new users may request connections.

There are many other scenarios from different domains—e.g., Facebook has on average 2 billion daily active users—approximately 23,000 users per second. These Facebook users can be divided into many groups (classes), and C-MaxRS can be used to retrieve the most interesting regions (with respect to particular requirements) among the active daily users. In this scenario, a large number of users can become online (*e*^+^), or go offline (*e*^−^) at almost-same time instant. Similarly, flocks of different kinds of animals may be approaching the water/food source; the containment of the diseases across the population and regions may vary; etc.

To address the efficiency of processing in such settings, we propose a novel technique, namely *C-MaxRS-Bursty*. The key idea of our approach is as follows: instead of processing every single update, we assume that the update streams are gathered for a period of time. Then, we create a modified slice-based index for the entire batch of the new events, and then snap the new data over the existing slice structure in a single pass. Finally, we perform the pruning conditions for each slice only once in an aggregated manner. Experimental results show that C-MaxRS-Bursty outperforms our one-at-a-time approach, C-MaxRS-DU, by a speed-up factor of 5–10.

The main contributions of this work can be summarized as follows:

- We formally define the C-MaxRS and C-MaxRS-DU problems (for both weighted and non-weighted versions) and provide a baseline solution using spatial subdivision (slices).
- We extend the solution to deal with spatial data streams (appearing and disappearing objects) for which we utilize effective pruning schemes for both appearing and disappearing events, capitalizing on a self-balancing binary search tree (e.g., AVL-tree) and a quad-tree.
- We propose an efficient methodology to handle bulk updates of data (i.e., updates with large data-volumes) along with the appropriate extensions of the data structures to cater to such settings.
- We demonstrate the benefits of our proposed method via experiments over a large dataset. Experiments over a wide range of parameters show that our approaches outperform the baseline algorithms by a factor of three to four. Moreover, experiments with bulk updates demonstrate the effectiveness and scalability of C-MaxRS-Bursty over other techniques (e.g., C-MaxRS-DU).

A preliminary version of this paper has appeared in Mostafiz et al. (2017), where we focused on non-weighted version of the C-MaxRS problem, i.e., we only count the number of objects inside the query window. We proposed two algorithms, *C-MaxRS*^+^ and *C-MaxRS*^−^ to efficiently solve C-MaxRS for data updates appearing and disappearing one at a time. The current article provides the following modifications and extensions to Mostafiz et al. (2017): (1) we provide the modified version of our algorithms from Mostafiz et al. (2017) to explicitly incorporate weighted version of the C-MaxRS problem, where each object and/or class can have different weights denoting its importance in the MaxRS computation. As it turns out (and demonstrated in the corresponding experiments) the weighted variant enables an increased pruning power; (2) we extend the work to consider novel settings of bulk updates handling of objects' appearance and disappearance and propose techniques for efficient computation of the C-MaxRS in those settings; (3) we conducted an extensive set of additional experiments to evaluate the benefits of our approaches.

In the rest of this paper, section 2 positions the work with respect to the existing literature, and section 3 formalizes the C-MaxRS problem. Section 4 describes the necessary properties of the conditional count functions and lays out the basic solution. Section 5 presents the details of our pruning strategies, along with the data structures and algorithms for incorporating dynamic data, while section 6 presents an extension of the C-MaxRS problem to include weights of objects (or, classes). Section 7 discusses the challenges of processing bursty inputs, and offers additional data structures and algorithms to deal with them. Section 8 presents the quantitative experimental analysis and Section 9 summarizes and outlines directions for future work.

## 2. Related Works

The Range Aggregation and Maximum Range Sum (MaxRS) queries, and their variants have been extensively studied in the recent years (e.g., Lazaridis and Mehrotra, 2001; Tao and Papadias, 2004; Cho and Chung, 2007; Sheng and Tao, 2011; Choi et al., 2014). A *Range Aggregation Query*, returning the aggregate result from a set of points, was solved for both 1-dimensional space—i.e., calculating result from set of values in given interval by Tao et al. (2014) and for 2 dimensional point space, i.e., calculating result from a given rectangle with fixed location by Papadias et al. (2001). To calculate the aggregate result, an *Aggregate Index*, storing the summarized result for specific region referenced by that index is used in Cho and Chung (2007). Different data structures are introduced to store the aggregate index—e.g., Lazaridis and Mehrotra (2001) proposed *Multi-Resolution Aggregate tree* (MRA-tree) to reduce the complexity. Although closely related, the MaxRS problem itself differs from these range aggregation queries.

The MaxRS problem was first addressed by researchers in the computational geometry community—e.g., Imai and Asano (1983) used a technique that finds connected components and a maximum clique of an intersection graph of rectangles in the plane. A solution based on plane sweep strategy was presented in Nandy and Bhattacharya (1995), where the input point-objects were “dualized” into rectangles (centered at the points and with dimensions equivalent to the query rectangle *r*). Then an interval tree was used to record the regions (a.k.a. windows) with highest number of intersecting (dual) rectangles along the sweep—denoting the possible locations for placing the (center of the) query rectangle, yielding O(nlogn) time complexity (*n* = number of points). However these solutions are not scalable, and Choi et al. (2014) proposed scalable extensions suited for LBS-applications—e.g., retrieve best location for a new franchise store with a specified delivery range. Subsequently, different variants of the MaxRS problem have been investigated:—constraining to underlying road networks (Phan et al., 2014; Zhou and Wang, 2016);—processing MaxRS queries in wireless sensor networks (Hussain et al., 2015; Wongse-ammat et al., 2017);—considering rotating MaxRS problem (Chen et al., 2015), where rectangles do not need to be axes parallel, i.e., allowing much more flexibility. A rather complementary work, tackling the problem of approximate solution to the MaxRS query was presented in Tao et al. (2013), using randomized sampling to bound the error with higher probability, with increasing number of objects in question. A more recent work, Liu et al. (2019) has proposed a novel solution PMaxRS to deal with the inherent location uncertainty of objects, and used smart candidate generation process (pruning) and sampling-based approximation algorithm (refinement) to efficiently solve the problem.

Monitoring MaxRS for dynamic settings, where objects can be inserted and/or deleted was first addressed in Amagata and Hara (2016). To efficiently detect the new locations for placing the query rectangle, Amagata and Hara (2016) exploited the aggregate graph *aG*2 in a grid index and devised a branch-and-bound algorithm (cf. Narendra and Fukunaga, 1977) over that *aG*2 graph for efficient approximation. We note that our work is complementary to Amagata and Hara (2016), in the sense that we addressed the settings of having different classes of objects and participation constraints based on them—whereas Amagata and Hara (2016) solves the basic MaxRS problem. Moreover, Amagata and Hara (2016) considered a sliding-window based model in the problem settings (i.e., if *m* new objects appear, then *m* old objects disappear in a time-window *T*), which is completely different to our event-based model. Additionally, we used contrasting approaches (and different data structures) in this work—dividing the 2D space into slices and slabs.

An interesting variant of MaxRS is addressed in Feng et al. (2016)—the, so called, *Best Region Search* problem, which generalizes the MaxRS problem in the sense that the goal of placing the query rectangle is to maximize a broader class of aggregate functions^3^. Our work adapts the concepts from Feng et al. (2016) (slices and pruning)—however, we tackle a different context: class-based participation constraints and dynamic/streaming data updates and, toward that, we also incorporated additional data structures (see section 5). As a summary, our methodology (as well as the actual implementation) is based on the idea of event driven approach for monitoring appearing and disappearing cases of objects, and we included a self-balancing binary tree (i.e., AVL-tree) to reduce the processing time that is needed for computing the MaxRS as per the event queue needs.

The issue of real-time query processing and indexing over spatio-temporal streaming data have been addressed extensively in prior literature, e.g., Hart et al., 2005; Mokbel et al., 2005; Dallachiesa et al., 2015, etc. For real-time computation, it is necessary to restrict the set of inspected data points at any time using techniques such as punctuation (embedded annotations), synopses (data summaries), windows (e.g., sliding windows—only items received in past *t* minutes), etc. In Mokbel et al. (2005), the authors implemented a continuous query processor designed specifically for highly dynamic environments. The proposed system utilized the idea of predicate-based sliding windows, and employed an incremental evaluation paradigm by continuously updating the query answer over a window. Dallachiesa et al. (2015) proposed both exact and approximate algorithms to manage *count-based uncertain sliding windows* for uncertain data streams (e.g., tuples can have both value and existential uncertainty). In contrast to these traditional window-based settings, we process C-MaxRS query in an event-based manner using all the data points received so far. This is necessary to maintain accurate answers for C-MaxRS over the whole dataset, i.e., trading off real-time processing power for accuracy.

On the other hand, both tree-based (cf. Hart et al., 2005) and grid-based (cf. Amini et al., 2011) indexing schemes have been proposed previously to deal with traditional streaming data. Dynamic Cascade Tree (DCT) is used in Hart et al. (2005) to index spatio-temporal query regions, ensuring optimized query processing for Remotely- Sensed Imagery (RSI) streaming data. Additionally, researchers such as Amini et al. (2011) have devised many hybrid clustering algorithms for data streams, using both density-based methods and grid-based indexing. In these density-based clustering algorithms, each point in a data-stream maps to a grid and grids are subsequently clustered based on their density. In our approach, we used slice-based (a specialized version of grid) indexing schemes to compute the range and class constrained optimal density clustering of data points (i.e., C-MaxRS).

Finally, as mentioned in section 1, a preliminary version of this work has been presented in Mostafiz et al. (2017). However, we note that the techniques for processing continuous monitoring queries over data streams (i.e., dynamic settings) must be adaptive, as data updates are often bursty and input characteristics may vary over time. Many previous works have demonstrated the tendency of bursty streams in various applications, and proposed general solutions such as Kleinberg (2003), Babcock et al. (2004), and Cervino et al. (2012), etc. For example, Babcock et al. (2004) utilized “load shedding” technique for aggregation queries over data updates, i.e., gracefully degrading performance when load is unmanageable; while Cervino et al. (2012) offered distributed stream processing systems to handle unpredictable changes in update rates. In this work, we address specifically the “algorithmic” part of the problem, i.e., presenting an optimal processing technique for C-MaxRS during bursty inputs. We conclude this section with a note that our proposed technique is implementation-independent, and can be augmented by existing distributed and parallel schemes seamlessly (cf. section 7).

## 3. Preliminaries

We now introduce the C-MaxRS problem and extend the definition for appearance of new objects, and disappearance of existing ones. Additionally, we discuss the concept of submodular monotone functions.

***C*-MaxRS &** ***C*-MaxRS-DU**: Let us define a set of *POIClass* *K* = {*k*_1_, *k*_2_, …, *k*_*m*_}, where each *k*_*i*_ ∈ *K* refers to a class (alternatively, tag and/or type) of the objects, a.k.a. points of interest (POI). In this setting, each object *o*_*i*_ ∈ *O* is represented as a *(location, class)* tuple at any time instant *t*. We denote a set *X*= {*x*_1_, *x*_2_, …, *x*_*m*_} as *MinConditionSet*, where |*X*| =|*K*| and each *x*_*i*_ ∈ ℤ+ denotes the desired lower bound of the number of objects of class *k*_*i*_ in the interior of the query rectangle *r*—i.e., the optimal region must have at least *x*_*i*_ number of objects of class *k*_*i*_. Let us assume *l*_*i*_ as the number of objects of type *k*_*i*_ in the interior of *r* centered at a point *p*. A utility function $f\left(O\right):P\left(O\right)\to {\mathbb{N}}_{0}$, mapping a subset of spatial objects to a non-negative integer is defined as below,

$$\begin{array}{l} f\left(O\right)=\left\{ \begin{array}{cc} \left(\sum_{i=1}^{\left|K\right|}l_{i}\right), & \text{if }\forall i\in \left\{1,2,3,...,|K|\right\},l_{i}>=x_{i}\\ 0, & \text{if }\exists i\in \left\{1,2,3,...,|K|\right\},l_{i}<x_{i} \end{array}\right. \end{array}$$

Additionally, we mark *O*_*r*_*p*__ as the set of spatial objects in the interior of rectangle *r* centered at any point *p*. Formally, **Conditional-MaxRS (C-MaxRS):** Given a rectangular spatial field 𝔽, a set of objects of interest *O* (bounded by 𝔽), a query rectangle *r* (of size *a* × *b*), a set of *POIClass* *K* = {*k*_1_, *k*_2_, …, *k*_*m*_} and a *MinConditionSet* *X* = {*x*_1_, *x*_2_, …, *x*_*m*_}, the C-MaxRS query returns an optimal location (point) p^*^ for *r* such that:

$$p^{*}=argmax_{p\in 𝔽}f\left(O_{r_{p}}\right)$$

where *O*_*r*_*p*__ ⊆ *O*.

Note that, in the case that there is no placement *p* for which all the conditions of *MinConditionSet* is met, the query will return an empty answer—indicating to the user to either increase the size of *R* or decrease the lower bounds for some classes.

In a spatial data stream environment, old points of interest may disappear and new ones may appear at any time instant. We can deal with this in two-ways:

- *Time-based:* C-MaxRS is computed on a regular time-interval δ.
- *Event-based:* C-MaxRS is computed on an *event*, where C-MaxRS is maintained (evaluated) every time a new point appears or an old point disappears.

Although faster algorithms can be developed in time-based settings, the solutions provided would be inherently erroneous for time between *t* and *t* + δ. On the other hand, event-based processing ensures that a correct answer-set is maintained all the time. Thus, we deal with the streaming data in event-based manner, for which we denote *e*^+^ as the new point *appearance* and *e*^−^ as the old point *disappearance* event. We note that, most of the settings for basic C-MaxRS remains same, except that the set of objects *O* is altered at each event. We define the set of points of interest in this data stream for any event *e*_*i*_ over an object *o*_*e*_*i*__ as:

$$\begin{array}{l} O_{e_{i}}=\left\{ \begin{array}{cc} O_{e_{i}-1}\cup \left\{o_{e_{i}}\right\}, & \text{if }e_{i}.type=e^{+}\\ O_{e_{i}-1}\backslash \left\{o_{e_{i}}\right\}, & \text{if }e_{i}.type=e^{-} \end{array}\right. \end{array}$$

Formally, **Conditional-MaxRS for Data Stream/Updates (C-MaxRS-DU)** definition is an extension of the above definition of C-MaxRS, for which we additionally have a sequence of events *E*={*e*_1_, *e*_2_, *e*_3_, …} where each *e*_*i*_ denotes the appearance or disappearance of a point of interest.

**Submodular Monotone Function:** Feng et al. (2016) devised solutions to a variant of the MaxRS problem (*best region search*) where the utility function for the given POIs is a submodular monotone function—which is defined as: [**Submodular Monotone Function**] If Ω is a finite set, a submodular function is a set function f:P(Ω)→ℝ if ∀_*X, Y*_⊂Ω, with *X* ⊆ *Y* and *x* ∈ Ω\*Y* we have (1) *f*(*X*∪{*x*}) − *f*(*X*) ≥ *f*(*Y*∪{*x*}) − *f*(*Y*) and (2) *f*(*X*) ≤ *f*(*Y*).

In the above definition, (1) represents the condition of submodularity, while (2) presents the condition of monotonicity of the function. In section 4, we will discuss these properties of our introduced utility function $f\left(O\right):P\left(O\right)\to {\mathbb{N}}_{0}$.

**Discussion:** Note that, for the sake of simplicity, initially we have considered only the counts of POIs when defining the utility function or conditions in *X*. In section 6, we show that they can be extended to incorporate different non-negative weights for objects with only minor modifications. Similarly, although in our provided examples, for brevity, we've only depicted one class per object, the techniques proposed in this work extends to the objects of multiple classes (or tags), e.g., objects can be considered as (*location, classes*) tuple.

## 4. Basic C-MaxRS

In this section, we first convert the C-MaxRS problem to its dual variant and then discuss important properties of the conditional weight function *f*(.), showing how we can utilize them to devise an efficient solution to process C-MaxRS.

### 4.1. C-MaxRS → Dual Problem

A naive approach to solve C-MaxRS is to choose each discrete point *p* iteratively from the rectangular spatial field 𝔽 and compute the value of *f*(*O*_*r*_*p*__) for the set of spatial objects covered by the query rectangle *r*. As there can be infinite number of points in 𝔽, this approach is too costly to be practical. Existing works (see Nandy and Bhattacharya, 1995; Feng et al., 2016; Hussain et al., 2017a) have demonstrated that feasible solutions can be derived for MaxRS (and related problems) by transforming it into its dual problem—*rectangle intersection problem*. A similar conversion is possible for C-MaxRS as well, enabling efficient solutions. In this regards, let *R*={*r*_1_, *r*_2_, …, *r*_*n*_} be a set of rectangles of user-defined size *a* × *b*. Each rectangle *r*_*i*_ ∈ *R* is centered at each point of interest *o*_*i*_ ∈ *O*, i.e., |*R*|=|*O*|. We define *r*_*i*_ as the *dual rectangle* of *o*_*i*_. Let us consider a function $g:P\left(R\right)\to {\mathbb{N}}_{0}$ that maps a set of dual rectangles to a non-negative integer. For a set of rectangles *R*_*k*_ = {*r*_1_, *r*_2_, …, *r*_*k*_}, let *g*(*R*_*k*_) = *f*({*o*_1_, *o*_2_, …, *o*_*k*_}). Note that, a rectangle is *affected* by a point *p* if it is in the interior of that rectangle. Let *A*(*p*) be the sets of rectangle affected by *p* ∈ 𝔽. Now, we can redefine C-MaxRS as the following equivalent problem:

*Given a rectangular spatial field* 𝔽*, a set of rectangles* *R=*{*r*_1_, *r*_2_, …, *r*_*n*_} *(with centers bounded by* 𝔽*) where each* *r*_*i*_ *is of a given size* *a* × *b, a set of* *POIClass* *K=*{*k*_1_, *k*_2_, …, *k*_*m*_} *and a* *MinConditionSet* *X=*{*x*_1_, *x*_2_, …, *x*_*m*_}*, retrieve an optimal location (point)* p^*^ *such that:*

$$p^{*}=argmax_{p\in P}g\left(A\left(p\right)\right),$$

*where* *A*(*p*) ⊆ *R*.

**Figure 2:**
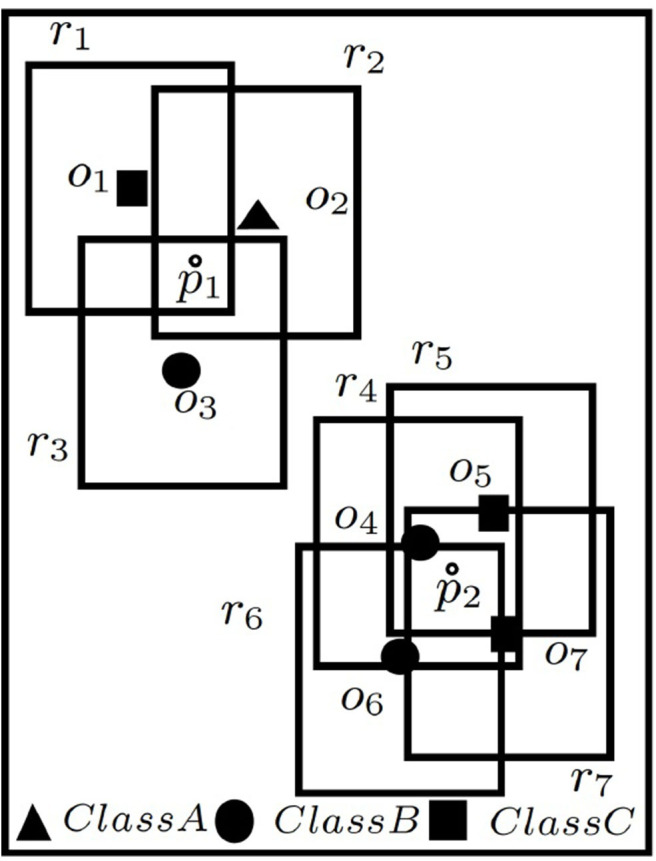
C-MaxRS → dual problem.

The bijection is illustrated with the help of Figure 2 using the same example (and conditions) of Figure 1, i.e., *the positioning of* *r* *must be such that at least 1 user is included from each of the classes A, B, and C, respectively*. Suppose, rectangles {*r*_1_, *r*_2_, *r*_3_, …, *r*_7_} are the dual rectangles of given objects {*o*_1_, *o*_2_, *o*_3_, …, *o*_7_} in Figure 2, and *p*_1_ and *p*_2_ are two points within the given space. *p*_1_ affects rectangles *r*_1_, *r*_2_, *r*_3_ and *p*_2_ affects *r*_4_, *r*_5_, *r*_6_, *r*_7_, i.e., *A*(*p*_1_) = {*r*_1_, *r*_2_, *r*_3_} and *A*(*p*_2_) = {*r*_4_, *r*_5_, *r*_6_, *r*_7_}. Thus, *g*(*A*(*p*_1_))=*f*({*o*_1_, *o*_2_, *o*_3_}) = 3 as the points conform to the constraints mentioned above, while *g*(*A*(*p*_2_))=*f*({*o*_4_, *o*_5_, *o*_6_, *o*_7_}) = 0 as they do not.

Similarly, C-MaxRS-DU can be redefined as follows:

*Given a rectangular spatial field* 𝔽*, a set of rectangles* *R=*{*r*_1_, *r*_2_, …, *r*_*n*_} *(with centers bounded by* 𝔽*) where each* *r*_*i*_ *is of a given size* *a* × *b, a set of* *POIClass* *K=*{*k*_1_, *k*_2_, …, *k*_*m*_}*, a* *MinConditionSet* *X=*{*x*_1_, *x*_2_, …, *x*_*m*_}*, and an event* *e* *(appearance/disappearance of a rectangle* *r*_*e*_*), update the optimal location (point)* p^*^ *such that:*

$$\begin{array}{l} p^{*}=argmax_{p\in P}g\left(A\left(p\right)\right), \end{array}$$

where

$$\begin{array}{l} A\left(p\right)\subseteq \left\{ \begin{array}{cc} R\cup \left\{r_{e}\right\}, & if\;e.type=e^{+}\\ R\backslash \left\{r_{e}\right\}, & if\;e.type=e^{-} \end{array}\right. \end{array}$$

### 4.2. Properties of *f* and *g*

A method to solve an instance of *Best Region Search* (BRS) problem was devised in Feng et al. (2016), where the weight function f:P(O)→ℝ is a submodular monotone function (cf. defined in section 3). In Feng et al. (2016), the problem is first converted to the dual *Submodular Weighted Rectangle Intersection (SIRI)* problem, and then optimization techniques are applied based on these properties of *f*(.). We now proceed to discuss submodularity and monotonicity of functions $f\left(O\right):P\left(O\right)\to {\mathbb{N}}_{0}$ and $g\left(R\right):P\left(R\right)\to {\mathbb{N}}_{0}$ in our problem settings. We establish two important results for *f* and *g* as follows:

Lemma 1. *Both *f* and *g* are monotone functions*.

*Proof*: For a set of spatial objects *O*,

$$\begin{array}{l} f\left(O\right)=\left\{ \begin{array}{cc} \left(\sum_{i=1}^{\left|K\right|}l_{i}\right), & \text{if }\forall i\in \left\{1,2,3,...,|K|\right\},l_{i}>=x_{i}\\ 0, & \text{if }\exists i\in \left\{1,2,3,...,|K|\right\},l_{i}<x_{i} \end{array}\right. \end{array}$$

For any of the classes, if the given lower-bound condition is not met, i.e. ∃*i* ∈ {1, 2, 3, ..., |*K*|}, *l*_*i*_ < *x*_*i*_, then *f*(*O*)=0 for the spatial object set *O*. However, if all of the conditions are satisfied—i.e., ∀*i* ∈ {1, 2, 3, ..., |*K*|}, *l*_*i*_ ≥ *x*_*i*_, then the utility value is equal to the number of spatial objects in *O*.

Let *O*_*i*_ ⊆ *O*_*j*_. If *O*_*i*_ = *O*_*j*_, *f*(*O*_*i*_) = *f*(*O*_*j*_), otherwise if *O*_*i*_⊂*O*_*j*_, there are three possible cases:

*Case (a)*: Both *O*_*i*_ and *O*_*j*_ fail to conform to the *MinConditionSet* *X*—then *f*(*O*_*i*_) = *f*(*O*_*j*_) = 0.

*Case (b)*: *O*_*j*_ conforms to *X*, but *O*_*i*_ does not—then *f*(*O*_*i*_) = 0 and *f*(*O*_*j*_) = |*O*_*j*_|. Thus, *f*(*O*_*i*_) < *f*(*O*_*j*_).

*Case (c)*: Both *O*_*i*_ and *O*_*j*_ conform to *X*, then *f*(*O*_*i*_) = |*O*_*i*_| and *f*(*O*_*j*_) = |*O*_*j*_|. As *O*_*i*_⊂*O*_*j*_, |*O*_*i*_| < |*O*_*j*_|, implying, *f*(*O*_*i*_) < *f*(*O*_*j*_).

We note that there are no possible cases where *O*_*i*_ conforms to *X*, but *O*_*j*_ does not. Thus, *f* is a monotone function. Let *R*_*i*_ and *R*_*j*_ be two sets of dual rectangles generated from the aforementioned two sets of spatial objects—*O*_*i*_ and *O*_*j*_, respectively. Here, *O*_*i*_ ⊆ *O*_*j*_ → *R*_*i*_ ⊆ *R*_*j*_. According to the definition of *g*, *g*(*R*_*i*_) = *f*(*O*_*i*_) and *g*(*R*_*j*_) = *f*(*O*_*j*_). As *f*(*O*_*i*_) ≤ *f*(*O*_*j*_), then *g*(*R*_*i*_) ≤ *g*(*R*_*j*_). Thus, *g* is a monotone function too.

Lemma 2. *None of *f* and *g* is a submodular function*.

*Proof*: Let us consider the settings of the preceding proof, i.e., two sets of spatial objects *O*_*i*_ and *O*_*j*_ (where *O*_*i*_ ⊆ *O*_*j*_), and corresponding sets of dual rectangles *R*_*i*_ and *R*_*j*_. Suppose, *O* and *R* are the set of all objects and dual rectangles, respectively. Let us consider a spatial object *o*_*k*_ ∈ *O*\*O*_*j*_ and its associated dual rectangle *r*_*k*_ ∈ *R*\*R*_*j*_. Then there is a possible case where *O*_*j*_ conforms to *X*, but neither *O*_*i*_ nor *O*_*i*_∪{*o*_*k*_} conform to *X*. As *O*_*j*_ conforms to *X*, *O*_*j*_∪{*o*_*k*_} will conform too. Thus, *f*(*O*_*i*_) = 0, *f*(*O*_*j*_) = |*O*_*j*_|, *f*(*O*_*i*_∪{*o*_*k*_}) = 0, *f*(*O*_*j*_∪{*o*_*k*_}) = |*O*_*j*_∪{*o*_*k*_}| = |*O*_*j*_|+1. Interestingly, we obtain: *f*(*O*_*i*_∪{*o*_*k*_}) − *f*(*O*_*i*_) = 0 − 0 = 0 and *f*(*O*_*j*_∪{*o*_*k*_}) − *f*(*O*_*j*_) = |*O*_*j*_|+1 − |*O*_*j*_| = 1; that means *f*(*O*_*i*_∪{*o*_*k*_}) − *f*(*O*_*i*_) < *f*(*O*_*j*_∪{*o*_*k*_}) − *f*(*O*_*j*_) violating the condition of submodularity. Hence, *f* is not submodular.

On the other hand, *g*(*R*_*i*_∪{*r*_*k*_}) − *g*(*R*_*i*_) = *f*(*O*_*i*_∪{*o*_*k*_}) − *f*(*O*_*i*_) = 0 − 0 = 0 and *g*(*R*_*j*_∪{*r*_*k*_}) − *g*(*R*_*j*_) = *f*(*O*_*j*_∪{*o*_*k*_}) − *f*(*O*_*j*_) = |*O*_*j*_|+1 − |*O*_*j*_| = 1; which means *g*(*R*_*i*_∪{*r*_*k*_}) − *g*(*R*_*i*_) < *g*(*R*_*j*_∪{*r*}) − *g*(*R*_*j*_). Thus, *g* is not submodular too.

Let us consider the example of Figure 2—suppose *O*_*i*_={*o*_4_, *o*_5_, *o*_6_, *o*_7_} and two new POIs *o*_8_ and *o*_9_ arrive from class *A* and *C*, respectively. let *O*_*j*_=*O*_*i*_∪{*o*_8_} (i.e., *O*_*i*_ ⊆ *O*_*j*_). Now, considering constraints for class A, B, and C, respectively, we have *f*(*O*_*i*_)=(0 + 2 + 2)(0)(1)(1)=0 and *f*(*O*_*j*_)=(1 + 2 + 2)(1)(1)(1)=5, i.e., *f*(*O*_*i*_) ≤ *f*(*O*_*j*_), proving monotonicity of *f*. But *f*(*O*_*i*_∪{*o*_9_})=(0 + 3 + 2)(0)(1)(1)=0 and *f*(*O*_*j*_∪{*o*_9_})=(1 + 3 + 2)(1)(1)(1)=6. Thus, (*f*(*O*_*i*_∪{*o*_9_}) − *f*(*O*_*i*_) = 0 − 0 = 0) < (*f*(*O*_*j*_∪{*o*_9_}) − *f*(*O*_*j*_) = 6 − 5 = 1), proving non-submodularity of *f*. Similar examples can be shown for *g* too.

### 4.3. Processing of C-MaxRS

Although *f* and *g* are not submodular functions, we show that their monotonicity property can be utilized to derive efficient processing and optimization strategies, similar to the ideas presented in Feng et al. (2016). For the rest of this section, let us denote *n* = |*O*| = |*R*|.

#### 4.3.1. Disjoint and Maximal Regions

**Figure 3:**
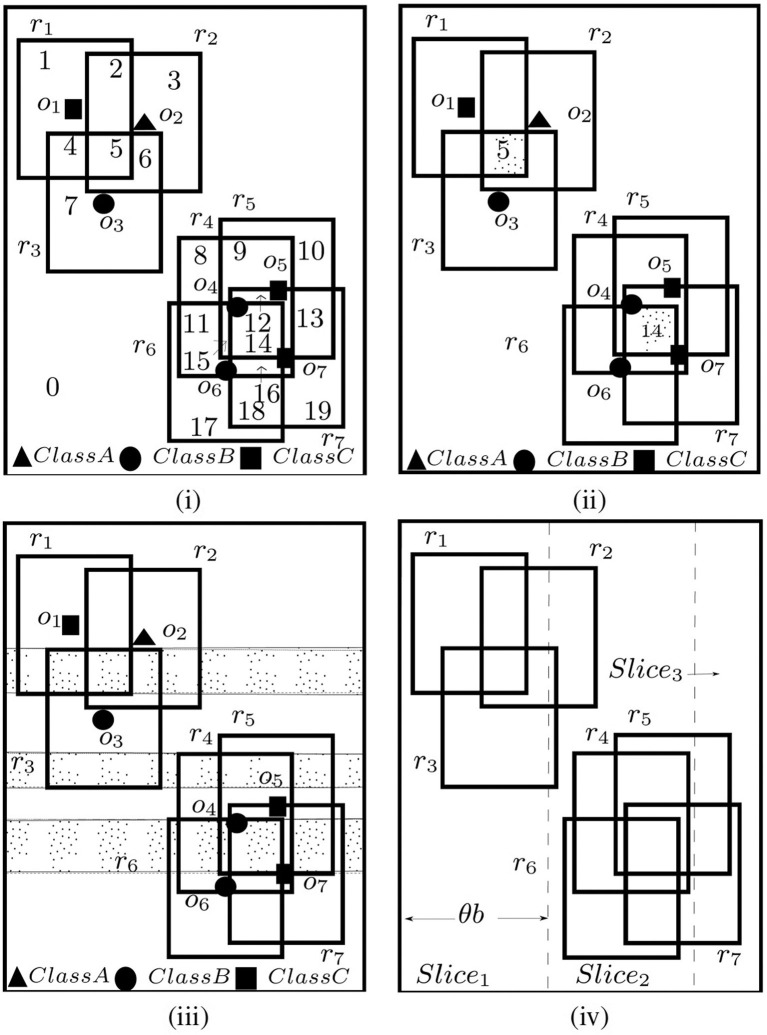
**(i)** Disjoint, **(ii)** Maximal regions, **(iii)** Maximal Slabs, and **(iv)** Slices.

The edges of the dual rectangles divide the given spatial field into *disjoint* regions where each disjoint region 𝔽_*d*_*i*__ is an intersection of a set of rectangles. Consider the examples shown in Figure 3i. Rectangles {*r*1, *r*2, ..., *r*7} divided the space into distinct regions numbered 0 − 19, e.g., region 0 is the region outside all rectangles, and region 14 is the intersection of rectangles {*r*4, *r*5, *r*6, *r*7}. Intuitively, all points in a single disjoint region 𝔽_*d*_*i*__ affects the same set of rectangles, i.e., *A*(*p*) is same for all *p* ∈ 𝔽_*d*_*i*__. There could be at most $O\left(n^{2}\right)$ disjoint regions (shown in Feng et al., 2016). To compute C-MaxRS, a straightforward approach can be to iterate over all the $O\left(n^{2}\right)$ disjoint regions (one point from each region) and choose the optimal one—thus reducing the search space into a finite point set. For example, we only need to evaluate 20 points for the settings of Figure 3i.

A disjoint region 𝔽_*d*_*i*__ is termed as a *maximal region* 𝔽_*m*_*i*__ if: (1) it is rectangular, and (2) its left, right, bottom, top edges are (respectively) the parts of the left, right, bottom and top edges of some dual rectangles of *R*. In Figure 3ii, region 5 and 14 are maximal regions. For example, the left, right, bottom, and top edges of region 5 is a part of the corresponding edges *r*_2_, *r*_1_, *r*_1_, *r*_3_ respectively. Feng et al. (2016) showed that for each distinct region 𝔽_*d*_*i*__, there exists a maximal region 𝔽_*m*_*i*__ such that *A*(𝔽_*d*_*i*__) ⊆ *A*(𝔽_*m*_*i*__). Using this idea, and the fact that *g*(.) is monotonic, we can shrink the possible search space to only the set of all maximal regions. As an example (see Figure 3), region 4 and 5 are affected by *R*_1_ = {*r*_1_, *r*_3_} and *R*_2_ = {*r*_1_, *r*_2_, *r*_3_}, respectively. As *R*_1_⊂*R*_2_, so by the monotonicity of *g*, *g*(*R*_1_) ≤ *g*(*R*_2_). So, only evaluating *g*(*R*_2_) is sufficient instead of evaluating both *g*(*R*_1_) and *g*(*R*_2_). Though there could still be $O\left(n^{2}\right)$ maximal regions in the worst case, the actual number in practice is much lower (compared to disjoint regions).

#### 4.3.2. Maximal Slabs and Slices

A *maximal slab* is the area between two horizontal lines in the space where the top line passes along the top edge of a dual rectangle and bottom one passes along the bottom edge of a dual rectangle, and the area between two horizontal lines contains no top or bottom edge of any other dual rectangles. In Figure 3iii, there are three maximal slabs, enclosed by the top and bottom edges of rectangles {*r*_3_, *r*_1_}, {*r*_4_, *r*_3_}, and {*r*_6_, *r*_5_} (top edges are solid line, and bottom edges are dotted lines). According to Feng et al. (2016), each maximal region intersects at least one maximal slab—i.e., the solution space can be reduced to the interior of all the maximal slabs only. As maximal slabs are defined based on one top and one bottom edge of dual rectangles, there could be at most O(n) maximal slabs.

All the maximal slabs can be retrieved using a horizontal sweep line algorithm in a bottom-up manner. A set is maintained to keep track of the rectangles intersecting the current slab, and a *flag* to indicate the type of the last horizontal edge processed. When the sweep line is at the bottom (top) edge of a rectangle, it is inserted into (deleted from) the set and *flag* is set to bottom (top). Additionally, when processing a top edge of a rectangle, the algorithm checks whether a maximal slab is encountered (i.e., currently *flag*=bottom). We can compute the upper bound for a slab by applying *g*(.) on the rectangles intersecting that slab, i.e., if *R*_*s*_*i*__ is the set of rectangles that intersects slab 𝔽_*s*_*i*__, then the upper bound of *g*(*p*) for any point *p* ∈ 𝔽_*s*_*i*__ is *g*(*R*_*s*_*i*__). For example, in Figure 3iii, {*r*_4_, *r*_5_, *r*_6_, *r*_7_} intersect the bottommost slab. So, the upper bound for that slab is *g*({*r*_4_, *r*_5_, *r*_6_, *r*_7_}) = 0 (as no members of class A present—not conforming to the introduced constraints in section 1).

Finally, the monotonicity of *g* allows us to adapt another optimization technique introduced in Feng et al. (2016)—*slices* (see Figure 3iv). The idea is to divide the whole space into vertical slices (along *x*-axis). The width of the slices is query-dependent, i.e., θ × *b*, where θ is a real positive constant value (θ > 1 and optimal value can be tuned empirically) and *b* is the width of the query rectangle *r*. After dividing the space into slices, we retrieve the slabs within each slice using the horizontal sweep-line algorithm described above and obtain upper-bound of a slice by computing the maximum upper-bound among all the slabs within that slice. We can then process the slices in a greedy manner—sort them in order of their upper-bounds and process one by one until the currently obtained result is greater than the upper-bounds of the remaining slices. Similar greedy approach can be adopted to process the maximal slabs within each slice. As an example, suppose there are four slices {*s*_1_, *s*_2_, *s*_3_, *s*_4_} with upper bounds {8, 3, 5, 2}, respectively. The order in which the slices will be processed is: {*s*_1_, *s*_3_, *s*_2_, *s*_4_}. Assume that after processing *s*_1_, current optimal *g* value is 3. So there is a possibility the optimal solution within *s*_3_ might exceed the current overall optimal solution of 3. After processing *s*_3_, if the result is 4, then processing *s*_2_ and *s*_4_ is unnecessary. Slices allow more pruning than slabs, and also O(n) maximal slabs is processed in all the slices (see Feng et al., 2016).

## 5. C-MaxRS in Data Updates

We now proceed with introducing novel techniques to deal with more realistic scenarios, i.e., data arriving in streams with the possibility of objects appearing and disappearing at different time instants. Using the approach of the basic C-MaxRS problem presented in previous section as a foundation, we augment the solution with compact data-structures and pruning strategies that enable effective handling of data streams environment.

### 5.1. Data Structures

Before proceeding with the details of the algorithms and pruning schemes, we describe the data structures used. We introduce two necessary data structures: quadtree (denoted *QTree*) and a self-balanced binary search tree (denoted *SliceUpperBoundBST*), and describe the details of our representation of slices. We re-iterate that while Feng et al. (2016) tackled the problem of best-placement with respect to an aggregate function, we are considering different constraints—class membership. In addition, we do not confine to a limited time-window. This is why, in addition to the quadtree used in Feng et al. (2016), we needed self-balancing binary tree to be invoked as dictated by the dynamics of the modifications.

#### 5.1.1. QTree

We need to process a large number of (variants of) range queries when computing *f* for any point, i.e., finding intersecting rectangles for a given rectangle. To ensure this is processed efficiently, we use quadtree (Samet, 1990)—a tree-based structure ensuring fast (O(logn)) insertion, deletion, retrieval and aggregate operations in 2D space. *QTree* recursively partitions 𝔽 into four equal sized rectangular regions until each leaf only contains one POI.

#### 5.1.2. *SliceUpperBoundBST*

Recall that the algorithm proposed in section 4.3 iterates through the slices in decreasing order of their maximum possible utility values (upper-bounds). Let us assume there are total *s* number of slices. To achieve this for basic C-MaxRS, sorting the slices in order is sufficient (O(slogs) operation). However, given the possibility of appearance (*e*^+^) and disappearance (*e*^−^) events in dynamic streaming scenarios, the upper-bounds of slices (and their respective order) may change frequently with time. To deal with these efficiently, we introduce a balanced binary search tree (*SliceUpperBoundBST*, see Nievergelt and Reingold, 1973) in our data structures instead of maintaining a sorted list whenever an event occurs. Different kinds of self-balancing binary search tree (e.g., AVL tree, Red-black tree, Splay tree, etc.) can be used for this purpose. We used AVL tree in our implementation. If there are ϵ number of dynamic events and *s* number of slices, sorting them on each event would incur a total of O((ϵ+1)slogs) time-complexity. Whereas we can build a balanced BST *SliceUpperBoundBST* initially in O(slogs), and update the tree at each event in O(logs) time. Thus the total cost of maintaining the sorted slices via *SliceUpperBoundBST* is O(slogs+ϵlogs) time. As in real-world applications running for a long time, we would incur large values of both ϵ and *s*, in which case, using *SliceUpperBoundBST* is much more efficient.

**Figure 4:**
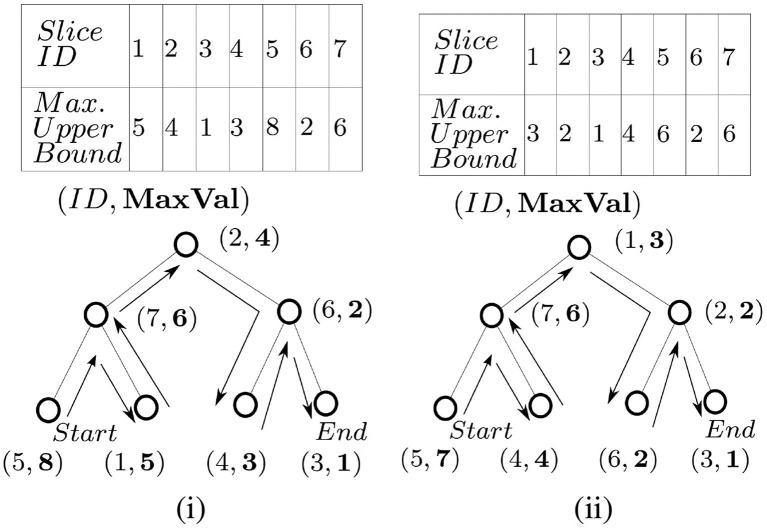
*SliceUpperBoundBST* at time **(i)** *t*_1_ and **(ii)** *t*_2_.

To traverse the slices in decreasing order via *SliceUpperBoundBST*, an in-order traversal from left to right order is needed (assuming, higher values are stored on the left children), and vice versa. *SliceUpperBoundBST* arranges the slices based on their upper bounds of *g*. In Figure 4, a sample slice structure (of 7 slices) and their respective maximum utility upper bounds (dummy values) are shown for two events at different times *t*_1_ and *t*_2_. The corresponding *SliceUpperBoundBST* structure for both cases is shown as well. The process of accessing the slices in decreasing order (an in-order traversal) is demonstrated in Figure 4ii.

#### 5.1.3. List of Slices

We use a list *S*_*slice*_ (where |*S*_*slice*_| = *s*) to maintain the slices and their related information. Each slice *s*_*i*_ ∈ *S*_*slice*_ is represented as a 6−tuple (*id, R, S*_*slabs*_, *p*_*c*_, *lazy, maxregsearched*). These fields are described as follows:

- ***id***: A numeric identification number for the slice.
- ***R***: The set of rectangles currently intersecting with the corresponding slice.
- ***S*_*slabs*_**: The set of maximal slabs in the interior of the slice.
- ***p*_*c*_**: The local optimum point within the slice.
- ***lazy***: This field is used to reduce computational overhead in certain scenarios. While processing streaming data, there are cases when an *e*^+^ or *e*^−^ event may alter the local solution (optimal point) for a particular slice, but overall, the global solution is guaranteed to remain unchanged. In those cases, we will not re-evaluate the local processing of that slice (i.e., pruning)—rather will set the *lazy* field to *true*. Later, when the possibility of a global solution change arises—local optimal points are re-processed for all the *lazy* marked slices to sync with the up-to-date state. Initially, *lazy* fields for all slices are set to *false*.
- ***maxregsearched***: This field is used to indicate whether the slice's local solution is up-to-date or not. *maxregsearched* is set to *true* when the corresponding slice is evaluated and its local maximal point is stored in *p*_*c*_. Initially, *maxregsearched* is set to *false* for all the slices. While processing C-MaxRS by iterating through the slices, all the slices with this field set to *true* are not re-evaluated (skipped).

### 5.2. Base Method

In this section, we start by introducing two related functions (sub-methods), and then proceed with describing the details of the base method to process C-MaxRS.

#### 5.2.1. PrepareSlices(*S*_*slice*_)

Function 1 takes *S*_*slice*_ as input and sets up different fields of each slice accordingly. For each slice *s*_*i*_ ∈ *S*_*slice*_, their respective *R* and *S*_*slabs*_ are computed (lines 2–3), and other variables are properly initialized (lines 4–6). In line 3, the maximum upper bounds of *g* (denoted *g*_*maxub*_) among all the slices is retrieved as well, while *ScanSlab* is the horizontal sweep-line procedure discussed in section 4.3.2. *SliceUpperBoundBST* is also build via line 7.

##### Time-Complexity

While analyzing time-complexities, we will denote |*S*_*slice*_| = *s* and number of rectangles (and objects too) as *n*. Suppose all of the slices in *S*_*slice*_ is passed to Function 1 for processing. In worst case scenario, line 2 takes O(n) time. Feng et al. (2016) shows *Scanslab*() (i.e., line 3) aggregately takes at most O(n) time for all the slices together. Any *SliceUpperBoundBST* operations (cf., line 4) need O(logs) time. Thus, the overall time-complexity of Function 1 is O(s(n+logs)+n)—or, O(sn) (as typically, *n* > *s*).

**Function 1:**
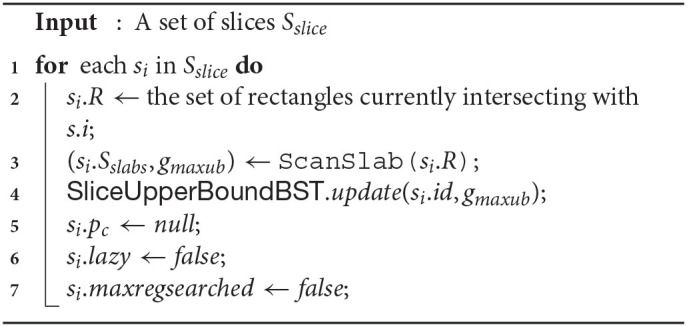
PrepareSlices(*S_slice_*)

#### 5.2.2. SliceSearchMR($p_{c}^{*}$)

Function 2 takes the current global maximal point $p_{c}^{*}$ as input and returns the updated solution. The function iterates through all the slices via in-order traversal of *SliceUpperBoundBST* from the *root* (lines 1–2). The process is terminated if *g*_*maxub*_ of the current slice is ≤ of current maximum utility value $g\left(A\left(p_{c}^{*}\right)\right)$ (lines 3–4), or when all the slices are evaluated. At each iteration, we check whether there exists an already computed solution (unchanged) for the slice. If so, we avoid recomputing it (lines 6–7), otherwise we retrieve the current optimal solution for the slice and update related variables accordingly (lines 9–11). Finally, we update the global optimal point by comparing it with the local solution (lines 12–13).

##### Time-Complexity

In the worst case scenario, all the nodes in *SliceUpperBoundBST* are traversed in Function 2. A stack based implementation of in-order traversal takes O(s) time, and computing the *g*() function can take up to O(n) time. Thus, the overall worst-case complexity for Function 2 is O(sn).

**Function 2:**
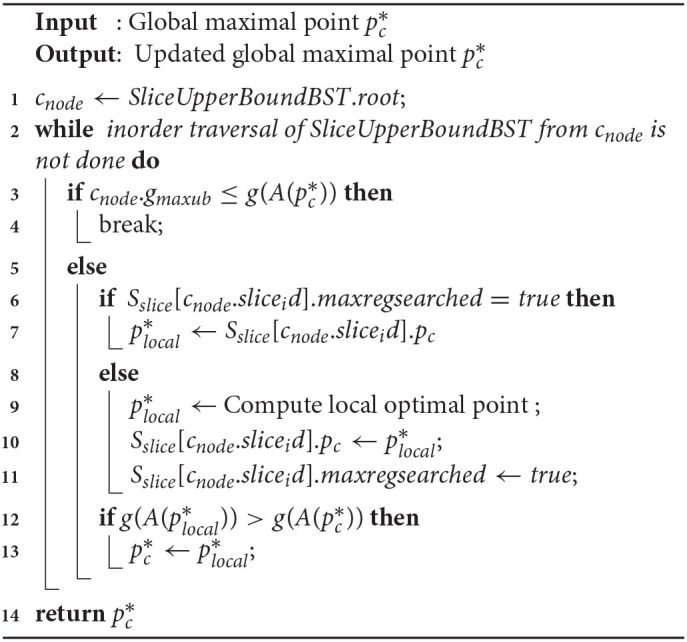
SliceSearchMR$\left(p_{c}^{*}\right)$

#### 5.2.3. SolveCMaxRS

Algorithm 1 presents the base method *SolveCMaxRS* that retrieves the optimal point $p_{c}^{*}$ from a snapshot of the database. $p_{c}^{*}$, *QTree* and *SliceUpperBoundBST* are initialized, and the dual rectangles of the given POIs *O* is computed in lines 1–4. In lines 5–6, we update the *QTree* by inserting all the dual rectangles in the structure. Line 7 retrieves the list of slices using the given width θ*b*. Finally, the method uses Function 1 to initialize the fields of slices properly in line 8, and computes the C-MaxRS solution using Function 2 in line 9.

##### Time-Complexity

Initializing and inserting all the rectangles in the quadtree takes O(nlogn) time along with a random initialization of *SliceUpperBoundBST* in O(s). Listing all the slices (line 7) also takes O(s) time. Using the complexities of *PrepareSlices*() and *SliceSearchMR*() from previous discussion, we can conclude that worst-case time complexity of Algorithm 1 is O(nlogn+sn).

**Algorithm 1:**
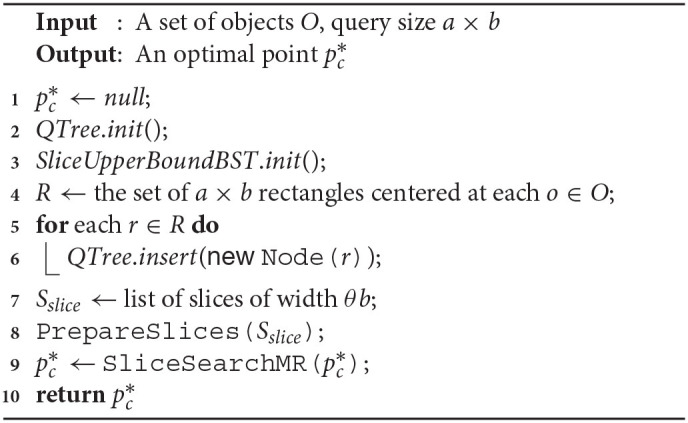
SolveCMaxRS(*O, a, b*)

### 5.3. Event-Based Pruning

Recall that, to cope with the challenges of real-time dynamic updates of the point space via data streams, we opted for the event-driven approach rather than the time-driven approach. Our goal is to maintain correct solution by performing instant updates during an event. In case of spatial data updates, a straightforward approach is to use Algorithm 1 whenever an event occurs. We now proceed to identify specific properties/states of events (both *e*^+^ and *e*^−^) that allow us to prune unnecessary computations while processing them. Note that, in this settings, a bunch of *e*^+^ and *e*^−^ events can occur at the same time.

#### 5.3.1. Pruning in *e*^−^

To derive an optimization technique for *e*^−^ events, let us first establish few related important results.

Lemma 3. *Removal of a rectangle* *r*_*e*_ *(object* *o*_*e*_) *from the point space* 𝔽 *never increases the value of* *g*(*A*(*p*)) *(correspondingly* *f*(*A*(*p*))), ∀*p* ∈ *P*.

*Proof*: Denote the removed rectangle as *r*_*e*_. We consider two cases:

- *r*_*e*_ ∈ *A*(*p*): After the removal of *r*_*e*_, the set of rectangles affected by *p* becomes *A*(*p*)\{*r*_*e*_}. Now, *A*(*p*)\{*r*_*e*_}⊂*A*(*p*). Hence, from Theorem 1, *g*(*A*(*p*)\{*r*_*e*_}) ≤ *g*(*A*(*p*)). Thus, the removal in this case does not increase *g*(*A*(*p*)).
- *r*_*e*_ ∉ *A*(*p*)): After removal of *r*_*e*_, the set of rectangles affected by *p* is still *A*(*p*). Hence, *g*(*A*(*p*)) remains unchanged. In this case as well, the removal does not increase *g*(*A*(*p*)).

Similarly, we can show a proof for removing an object—i.e., *o*_*e*_ from 𝔽.

Lemma 3 paves the way for the pruning of slices from being considered a solution at *e*^−^ events.

Lemma 4. *The maximum utility point (global solution)* $p_{c}^{*}$ *is unchanged after the removal of a rectangle* *r*_*e*_ *from the space* 𝔽 *if* $r_{e}\notin A\left(p_{c}^{*}\right)$.

*Proof*: Here, $r_{e}\notin A\left(p_{c}^{*}\right)$. Suppose, after removing *r*_*e*_, $A^{\prime }\left(p_{c}^{*}\right)$ rectangles are affected by $p_{c}^{*}$. Note that, $A^{\prime }\left(p_{c}^{*}\right)$=$A\left(p_{c}^{*}\right)$ (as $r_{e}\notin A\left(p_{c}^{*}\right)$), implying $g\left(A^{\prime }\left(p_{c}^{*}\right)\right)=g\left(A\left(p_{c}^{*}\right)\right)$. Thus, the utility values of $p_{c}^{*}$ remains the same. By Lemma 3, the removal of *r*_*e*_ does not increase the utility value of *p*, ∀*p* ∈ *P*. Suppose, the utility value of a point *p*, (*p* ∈ *P* and *p* ≠ *p*_*c*_), are *g*(*A*(*p*)) and *g*′(*A*(*p*)), respectively before and after the removal of *r*_*e*_, then *g*′(*A*(*p*)) ≤ *g*(*A*(*p*)). Again, $p_{c}^{*}$ being the maximal point, $g\left(A\left(p\right)\right)\leq g\left(A\left(p_{c}^{*}\right)\right)$, $\forall p\in P,p\neq p_{c}^{*}$. Above mentioned inequalities imply that $g^{\prime }\left(A\left(p\right)\right)\leq g\left(A^{\prime }\left(p_{c}^{*}\right)\right)$, $\forall p\in P,p\neq p_{c}^{*}$, meaning $p_{c}^{*}$ remains unchanged.

Using Lemma 4, we can prune local slice processing at an *e*^−^ event, if $r_{e}\notin A\left(p_{c}^{*}\right)$, i.e., we need to only update *QTree* in this case.

Lemma 5. *The utility value of the maximal point* $p_{c}^{*}$ *is changed after the removal of a rectangle* *r*_*e*_ if $r_{e}\in A\left(p_{c}^{*}\right)$.

*Proof*: If $p_{c}^{*}$ is returned as the maximal point, then $g\left(A\left(p_{c}^{*}\right)\right)>0$ (i.e., we have a solution). After the removal of *r*_*e*_, the set of rectangles affected by $p_{c}^{*}$ becomes $A\left(p_{c}^{*}\right)-\left\{r_{e}\right\}$. There are two possible cases:

- $A\left(p_{c}^{*}\right)-\left\{r_{e}\right\}$ conforms to *X*: In this scenario, $g\left(A\left(p_{c}^{*}\right)\right)-g\left(A\left(p_{c}^{*}\right)-\left\{r_{e}\right\}\right)=|A\left(p_{c}^{*}\right)|-\left(|A\left(p_{c}^{*}\right)|-1\right)=1$.
- $A\left(p_{c}^{*}\right)-\left\{r_{e}\right\}$ does not conform to *X*: Here, $g\left(A\left(p_{c}^{*}\right)\right)-g\left(A\left(p_{c}^{*}\right)-\left\{r_{e}\right\}\right)=|A\left(p_{c}^{*}\right)|-0=|A\left(p_{c}^{*}\right)|$.

In both cases, $g\left(A\left(p_{c}^{*}\right)\right)$ is changed.

Lemma 5 implies that, if a rectangle removed at an *e*^−^ event is in $A\left(p_{c}^{*}\right)$, we need to re-evaluate local solutions for the respective slice(s), and update global maximal point if necessary.

Lemma 6. *Suppose a point space* *P* *is divided into a set of slices* *S*_*slice*_, *and the slice containing the maximum utility point* $p_{c}^{*}$ *is* *s*_*max*_. *Let*, *S*_*s*_ *be another set of slices, where* *S*_*s*_⊂*S*_*slice*_ *and* *s*_*max*_ ∉ *S*_*s*_. *Subsequently, the removal of a rectangle* *r*_*e*_ *spanning through only the slices in* *S*_*s*_, *i.e., affecting only the local maximum utility values of* *s*_*i*_, ∀*s*_*i*_ ∈ *S*_*s*_, *does not have any effect on the global maximum utility point* $p_{c}^{*}$.

*Proof*: Let $p_{local}^{*}$ be the maximum utility point of a slice *s*_*i*_ ∈ *S*_*s*_. ∀*p* ∈ *s*_*i*_ where *s*_*i*_ ∈ *S*_*s*_, $g\left(A\left(p_{c}^{*}\right)\right)\geq g\left(A\left(p_{local}^{*}\right)\right)$ and $g\left(A\left(p_{local}^{*}\right)\right)\geq g\left(A\left(p\right)\right)$. According to Lemma 3, after the removal of *r*_*e*_, for any *s*_*i*_ ∈ *S*_*s*_, *g*(*A*(*p* − {*r*_*e*_})) ≤ *g*(*A*(*p*)). From the above three inequalities, we can deduce: ∀*p* ∈ *s*_*i*_ where *s*_*i*_ ∈ *S*_*s*_, $g\left(A\left(p\right)-\left\{r_{e}\right\}\right)\leq g\left(A\left(p_{c}^{*}\right)\right)$. This holds true ∀*s*_*i*_∈*S*_*s*_. Thus, $p_{c}^{*}$ still remains the maximum utility point (as *s*_*max*_ is not altered), and *s*_*max*_ is still the slice containing $p_{c}^{*}$.

Lemma 6 implies that, if the slice containing global maximal point $p_{c}^{*}$ is unchanged while some other slices are altered, then following the update of *QTree*, we can delay the processing of altered slices at that time instance as it is not going to affect the global maximal answer anyway. For this reason, we incorporated the *lazy* field in each slice. In this case, we set *lazy* to *true* for each of these altered slices, indicating that they should be re-evaluated later only when the slice containing global maximal point is altered.

#### 5.3.2. Pruning in *e*^+^

During an *e*^+^ event, a rectangle (object) appears in the given space 𝔽. We now present two lemmas, based on which we derive pruning strategies at *e*^+^ events.

Lemma 7. *Addition of a rectangle* *r*_*e*_ *(object* *o*_*e*_) *in the given space 𝔽 never decreases the value of* *g*(*A*(*p*)) *(correspondingly* *f*(*A*(*p*))), ∀*p* ∈ *P*.

*Proof*: Let the added rectangle be *r*_*e*_. We consider two cases:

- *r*_*e*_ ∈ *A*(*p*): After the addition of *r*_*e*_, the set of rectangles affected by *p* becomes *A*(*p*)∪{*r*_*e*_}. Now, *A*(*p*)⊂*A*(*p*)∪{*r*_*e*_}. Hence, from Theorem 1, *g*(*A*(*p*)∪{*r*_*e*_}) ≥ *g*(*A*(*p*)). So, in this case *g*(*A*(*p*)) does not decrease.
- *r*_*e*_ ∉ *A*(*p*)): After addition of *r*_*e*_, the set of rectangles affected by *p* still remains *A*(*p*). Hence, *g*(*A*(*p*)) does not change as well. Thus, *g*(*A*(*p*)) does not decrease in this scenario as well.

Similarly, we can show a proof for adding an object—i.e., *o*_*e*_ to 𝔽.

For *e*^−^ events, we leveraged on ideas like Lemma 3—i.e., removal of a rectangle never increases utility value of a point, to devise clever pruning schemes depending on the fact that local or global maximal points are guaranteed to be unchanged in certain scenarios. But, for *e*^+^ events, those are not applicable as addition of a rectangle *may* increase utility of affected points. Interestingly, though, there are scenarios when the utility values are unchanged, e.g., when *A*(*p*) does not conform to *X*. Also, as shown in the 2nd case of the proof of Lemma 7—we only process a slice if its affected by the addition of *r*_*e*_.

Lemma 8. *Suppose, we have a set of classes* *K* = {*k*_1_, *k*_2_, …, *k*_*m*_}, *and are given corresponding* *MinConditionSet* *X* = {*x*_1_, *x*_2_, …, *x*_*m*_}. *Let* *R* *be the set of rectangles overlapping with a slice* *s*_*i*_ ∈ *S*_*slice*_, *and let* *l*_*i*_ *be the number of rectangles of class* *k*_*i*_ in *R*. *Then, addition of a rectangle* *r*_*e*_ *of class* *k*_*i*_ *has no effect on the local maximal solution of* *s*_*i*_ *if:*

*(1)* *x*_*i*_ − *l*_*i*_ ≥ 2, or
*(2)* (∃*l*_*j*_ ≠ *l*_*i*_) *x*_*j*_ − *l*_*j*_ ≥ 1

*Proof*: (1) In this settings, the maximum possible utility value of *s*_*i*_ before addition of *r*_*e*_ is 0. Because, even if for a point *p* ∈ *s*_*i*_, *A*(*p*) = *R*, then *g*(*A*(*p*))=0 as *l*_*i*_ < *x*_*i*_ and *R* does not conform to *X*. After the addition of *r*_*e*_, suppose the number of class *k*_*i*_ objects in *R* is $l_{i}^{\prime }$, i.e., $l_{i}^{\prime }$=*l*_*i*_ + 1. As given *x*_*i*_ − *l*_*i*_ ≥ 2, then $l_{i}^{\prime }<x_{i}$. Thus, *R* still does not conform to *X*, and maximum possible utility value of *s*_*i*_ remains 0.

(2) Similarly, the maximum possible utility value of *s*_*i*_ before addition of *r*_*e*_ is 0. Because, even if for a point *p* ∈ *s*_*i*_, *A*(*p*) = *R*, then *g*(*A*(*p*))=0 as *l*_*j*_ < *x*_*i*_ for ∃*l*_*j*_ ≠ *l*_*i*_, and *R* does not conform to *X*. After the addition of *r*_*e*_ of class *k*_*i*_, *l*_*j*_ remains unchanged. Thus, *R* still does not conform to *X*, and maximum possible utility value of *s*_*i*_ remains 0.

Lemma 8 lays out the process of pruning during an *e*^+^ event. For each slice, we maintain an integer value *diff* (i.e., *x*_*i*_ − *l*_*i*_) per class in *K* denoting whether the corresponding upper-bound for that class has been met or not. When adding a rectangle of class *k*_*i*_, for each affected slices, we first check whether *diff*_*i*_ ≥ 2, and if so—we just update *diff*_*i*_ and skip processing that slice. Similarly, if *diff*_*i*_ ≤ 1, but for ∃*diff*_*j*_ ≥ 1, we can skip the slice. For example, suppose we have a setting of three classes *A*, *B*, *C* where *X*={2, 3, 5}. Suppose a slice contains {2, 1, 4} members of respective classes. In this case, arrival of a rectangle of class *B* or *C* has no effect on that slice. We incorporate these ideas in our Algorithm 3 (although, for brevity, we skip details of implementing and maintaining *diff* in algorithms).

### 5.4. Algorithmic Details

We now proceed to augment the ideas from the previous section in our base solution. We provide the details of two algorithms *SolveCMaxRS*^−^ and *SolveCMaxRS*^+^, implementing the ideas of pruning in *e*^−^ and *e*^+^ events, respectively.

**Algorithm 2:**
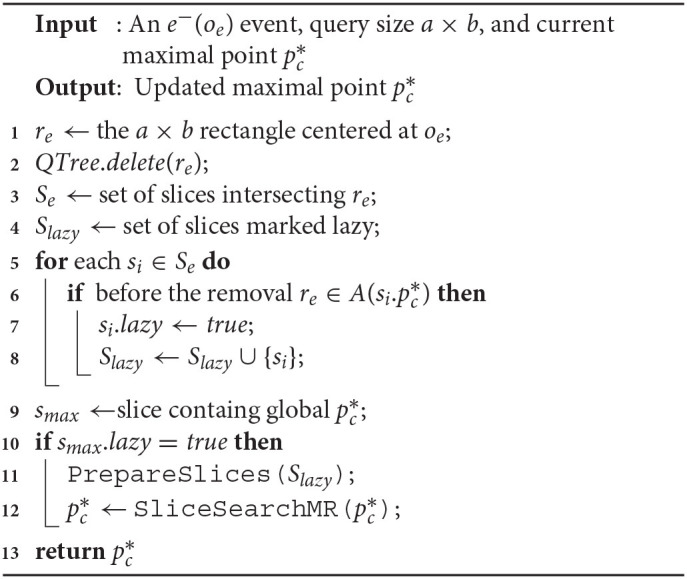
SolveCMaxRS${}^{-}\;\left(e^{-}\left(o_{e}\right),a,b,p_{c}^{*}\right)$

#### 5.4.1. *SolveCMaxRS*^−^

In Algorithm 2, we present the detailed method for maintaining C-MaxRS result during an *e*^−^ event using the ideas introduced in section 5.3.1. Firstly, *r*_*e*_ is retrieved (from *o*_*e*_) and then deleted from then *QTree* is updated accordingly (cf. lines 1–2). Subsequently, in lines 3–4, all the slices intersecting with *r*_*e*_ is retrieved and the set of slices marked lazy (*S*_*lazy*_) is initialized. Lines 5-8 iterate through all the affected slices one by one and check for each of them to see if the local maximal point $s_{i}.p_{c}^{*}$ is affected by *r*_*e*_—if so, it marks them as lazy for future update and also adds them to *S*_*lazy*_. If the slice containing global maximal point i(i.e., *s*_*max*_) is not affected, then the processing of slices in *S*_*lazy*_i skipped (pruning) in lines 9–12. Otherwise, if pruning is not possible, necessary computations are carried out in lines 11–12.

##### Time-Complexity

Deleting from a quadtree takes O(logn) time (line 2). Listing all the intersecting and lazy slices in worst cases will generate O(s) computations (lines 3–4). Iterating over all the overlapped slices and computing *g*() takes up O(sn) times in worst case (lines 5–8). If pruning is not possible, the complexities of *PrepareSlices*() and *SliceSearchMR*() adds up too (lines 10-12). The overall worst-case time complexity of Algorithm 2 is O(sn+s+logn+sn+sn)—or, in short, O(sn).

#### 5.4.2. *SolveCMaxRS*^+^

In Algorithm 3, we initially retrieve the dual rectangle *r*_*e*_ associated with the event and update *QTree* by inserting *r*_*e*_ as a new node in lines 1–2. Then, the set of slices affected by *r*_*e*_ is computed and *S*_*lazy*_ is initialized in lines 3–4. We introduce a Boolean variable *isPrunable* in line 5 to track whether Lemma 8 can be applied or not. Lines 6-10 iterate through all the affected slices one by one, an checks: if *s*_*i*_.*R* now conforms to *X* and makes change accordingly (modifies *isPrunable*), and sets up *s*_*i*_.*lazy* and list *S*_*lazy*_ properly. Lines 11–12 prunes the event if conditions of Lemma 8 is satisfied, i.e., if *isPrunable* = *true* then the global maximal $p_{c}^{*}$ needs no update. Otherwise, it processes C-MaxRS on the snapshot (lines 13–14).

##### Time-Complexity

The analysis of lines 1–4 here is similar to Algorithm 2. Iterating over all the intersecting slices and checking the constraints takes up O(s×|X|) times in worst case. So, if pruning is possible, the time-complexity of Algorithm 3 is O(s×|X|+s+logn) time (faster than pre-pruning stage of Algorithm 2). But, in worst case, if pruning is not possible, then the complexity will be O(sn) (similar to Algorithm 2).

**Algorithm 3:**
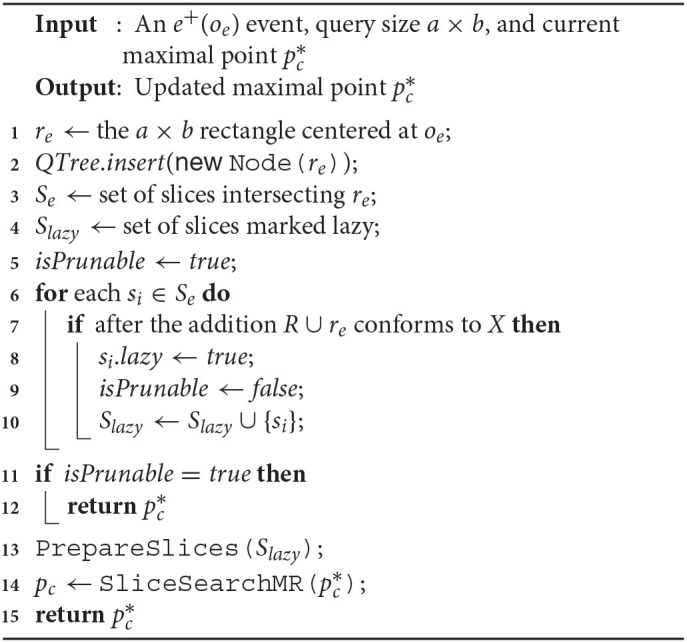
SolveCMaxRSSolveCMaxRS^+^ $\left(e^{+}\left(o_{e}\right),a,b,p_{c}^{*}\right)$

## 6. Weighted C-MaxRS

In the discussions so far, we only considered the counting variant of the C-MaxRS problem, i.e., the weights of each participating object are all equal to 1. While we have noted the portability of the results, in this section, we explicitly show how the algorithms and pruning schemes proposed thus far should be modified to cater to the case when the objects can have different weights. Firstly, we appropriately revise the definitions of *f*, *g*, and C-MaxRS-DU to allow different weights, and show that it does not affect the monotonicity and non-submodularity of *f* and *g*. Subsequently, we outline the modifications for the pruning schemes for the weighted version. While there are no major changes incurred in the fundamental algorithmic aspects, we note that weights may have impact on the pruning effects, as illustrated in section 8.

### 6.1. Redefining *f*, *g*, and C-MaxRS-DU

***f*^*w*^**: Let us define a set of *POIClass* *K* = {*k*_1_, *k*_2_, …, *k*_*m*_}, where each *k*_*i*_ ∈ *K* refers to a class of objects. Suppose, *O* = {*o*_1_, *o*_2_, …, *o*_*n*_} is the set of objects (POIs), and the set *W* = {*w*_1_, *w*_2_, …, *w*_*n*_}, where *w*_*i*_ > 0, ∀*w*_*i*_ ∈ *W*, contains the weight values of all POIs, i.e., the weight of an object *o*_*i*_ is *w*_*i*_. In this setting, each object *o*_*i*_ ∈ *O* is represented as a *(location, class*, *w*_*i*_*)* tuple at any time instant *t*. We denote a set *X*= {*x*_1_, *x*_2_, …, *x*_*m*_} as *MinConditionSet*, where |*X*| =|*K*| and each *x*_*i*_ ∈ ℝ+ denotes the desired lower bound of the weighted-sum of the objects of class *k*_*i*_ in the interior of the query rectangle *r*, i.e., $\overset{\forall o_{i}}{\underset{o_{i}\in r\wedge o_{i}.class=k_{i}}{\sum }}w_{i}$. Thus, the optimal region must have objects of class *k*_*i*_ whose weights add up to at least *x*_*i*_. Let us define $l_{i}^{w}$, a non-negative real number, for a given set of objects *O* as follows:

$$\begin{array}{l} l_{i}^{w}=\overset{\forall o_{j}}{\underset{o_{j}\in O\wedge o_{j}.class=k_{i}}{\sum }}w_{j}. \end{array}$$

Subsequently, we can define a utility function $f^{w}\left(O\right):P\left(O\right)\to {\mathbb{N}}_{0}$, mapping a subset of spatial objects to a non-negative real number as below,

$$\begin{array}{l} f^{w}\left(O\right)=\left\{ \begin{array}{cc} \left(\sum_{i=1}^{\left|K\right|}l_{i}^{w}\right), & \text{if }\forall i\in \left\{1,2,3,...,|K|\right\},l_{i}^{w}>=x_{i}\\ 0, & \text{if }\exists i\in \left\{1,2,3,...,|K|\right\},l_{i}^{w}<x_{i}. \end{array}\right. \end{array}$$

**C-MaxRS-DU**: Let us denote the rectangle *r* centered at point *p* as *r*_*p*_, and *O*_*r*_*p*__ as the set of spatial objects in the interior of *r*_*p*_. We can now define C-MaxRS-DU as follows (including the weights):**Conditional-MaxRS for Data Updates (C-MaxRS-DU):** Given a rectangular spatial field 𝔽, a set of objects of interests *O* (bounded by 𝔽) and their corresponding set of weight values *W*, a query rectangle *r* (of size *a* × *b*), a set of *POIClass* *K* = {*k*_1_, *k*_2_, …, *k*_*m*_}, a *MinConditionSet* *X* = {*x*_1_, *x*_2_, …, *x*_*m*_}, and a sequence of events *E*={*e*_1_, *e*_2_, *e*_3_, …} (where each *e*_*i*_ denotes the appearance or disappearance of a point of interest), the C-MaxRS-DU query maintains the optimal location (point) p^*^ for *r* such that:

$$p^{*}=argmax_{p\in 𝔽}f^{w}\left(O_{r_{p}}\right)$$

where *O*_*r*_*p*__ ⊆ *O*_*e*_ for every event *e* in *E* of the data stream.

***g*^*w*^**: Similar to the function *g*, we can introduce *g*^*w*^ as a bijection of *f*^*w*^, i.e., for a set of rectangles *R*_*k*_ = {*r*_1_, *r*_2_, …, *r*_*k*_}, let $g^{w}\left(R_{k}\right)=f^{w}\left(\left\{o_{1},o_{2},\ldots ,o_{k}\right\}\right)$. $g^{w}:P\left(R\right)\to {\mathbb{R}}_{0}$ maps a set of dual rectangles to a non-negative real number (weighted-sum).

### 6.2. Monotonicity and Non-submodularity of *f*^*w*^ and *g*^*w*^

As we define *w*_*i*_ ∈ *W* as a positive real number, the weighted-sum of a set of objects—$\underset{o_{i}}{\sum }w_{i}$, is also a positive real number. This is similar to the counting variant of the problem. Thus, using the similar logic as Lemma 1 and Lemma 2, we derive the following:

Lemma 9. *Both *f*^*w*^ and *g*^*w*^ are monotone functions*.

Lemma 10. *None of *f*^*w*^ and *g*^*w*^ is a submodular function*.

The proofs follow the similar intuition as the corresponding proofs of Lemma 1 and Lemma 2 and are omitted –however, we proceed with discussing their implication in a more detailed manner next.

### 6.3. Discussion

Lemma 9 and Lemma 10 show that the properties of the utlity functions remain same, for both counting and weighted version. Subsequently, we can derive the following:

Lemma 11. *Removal of a rectangle* *r*_*e*_ *(object* *o*_*e*_) *from the point space* 𝔽 *never increases the value of* *g*^*w*^(*A*(*p*)) *(correspondingly* *f*^*w*^(*A*(*p*))), ∀*p* ∈ *P*.

Lemma 11 can be proved in similar way as the proof of Lemma 3, as *f*^*w*^ and *g*^*w*^ are also monotonous. Thus, Lemma 11 validates the other necessary lemmas (i.e., Lemma 4, 5, and 6) related to the *e*^−^ pruning scheme. This shows that we can solve the problem of an *e*^−^ event, for an object *o*_*e*_ (rectangle *r*_*e*_) and its weight *w*_*e*_, by using the same algorithm *SolveCMaxRS*^−^. For the *e*^+^ event, we present the following lemmas: (skipping proof for brevity)

Lemma 12. *Addition of a rectangle* *r*_*e*_ *(object* *o*_*e*_) *in the given space* 𝔽 *never decreases the value of* *g*^*w*^(*A*(*p*)) (*correspondingly* *f*^*w*^(*A*(*p*))), ∀*p* ∈ *P*.

Lemma 13. *Suppose, we have a set of classes* *K* = {*k*_1_, *k*_2_, …, *k*_*m*_}, *and are given corresponding* *MinConditionSet* *X* = {*x*_1_, *x*_2_, …, *x*_*m*_}. *Let* *R* *be the set of rectangles overlapping with a slice* *s*_*i*_ ∈ *S*_*slice*_, *and let* $l_{i}^{w}$ *be the weighted-sum of rectangles of class* *k*_*i*_ *in* *R*. *Then, addition of a rectangle* *r*_*e*_ *of class* *k*_*i*_ *has no effect on the local maximal solution of* *s*_*i*_ *if:*

*(1)* $x_{i}-l_{i}^{w}>w_{e}$, or
*(2)* (∃*l*_*j*_ ≠ *l*_*i*_) $x_{j}-l_{j}^{w}>0$

Lemmas 12 and 13 demonstrates that an *e*^+^ event, for an object *o*_*e*_ (rectangle *r*_*e*_) and its weight *w*_*e*_, can be processed similarly via *SolveCMaxRS*^+^ algorithm.

## 7. C-MaxRS in Bursty Updates

In many spatial applications, the data streaming rate often varies wildly depending on various external factors—e.g., the time of the day, the need of the users, etc. A peculiar phenomenon in such cases is the, so called, bursty streaming updates—which is, the streaming rate becomes unusually high and a large number of objects appearing or disappearing in a very short interval. In such scenarios, instead of processing every single update, we assume that the update streams are gathered for a period of time. The C-MaxRS-DU algorithm is based on sequential processing of events, and thus, its efficiency is particularly sensitive to the bursty input scenario. In this section, we first briefly discuss the challenges of processing bulk of events via Algorithm 2 and 3, and argue that a different technique is necessary. Subsequently, we propose additional data-structures and a new algorithm, C-MaxRS-Bursty, to maintain C-MaxRS during bursty streaming updates scenarios. Finally, we briefly discuss how our proposed scheme can be utilized in a distributed manner, for the purpose of further improvements in scalability.

### 7.1. Challenges

As per the algorithms presented in section 5, Algorithm 2 (*SolveCMaxRS*^−^) and Algorithm 3 (*SolveCMaxRS*^+^) are used to deal with any new *e*^−^ and *e*^+^ event, respectively. The worst case time complexity of both the algorithms is O(sn). Let us denote γ as the average streaming (a.k.a. bulk-updates) rate during a bursty stream scenario, i.e., γ events (both *e*^+^ and *e*^−^) occur simultaneously per time instance. In this setting, the worst-case complexity of processing these events using C-MaxRS-DU is O(γsn). We note that, due to the effectiveness of the pruning schemes, the average processing time is considerably faster than the worst case complexity presented here (details in section 8). However, the overhead of performing Algorithm 2 and Algorithm 3 γ times is still significant, specially when fast and accurate responses are required. For example, line 3 of Algorithm 2 takes O(s) time to find the slices *S*_*e*_ that intersect with the new event rectangle *r*_*e*_. Instead of computing this γ times (i.e., γ×O(s)), it would be better if we scan the list of slices only once, and retrieve all the slices that are affected by the new γ events in one pass. Moreover, if the slice containing global $p_{c}^{*}$, i.e., *s*_*max*_, is affected by multiple events, then *PrepareSlices*() and *SliceSearchMR*() would be redundantly processed multiple times. Hence, the intuition is that we can get rid of these overheads by dealing with the bursty events aggregately.

To this end, we propose an additional data structure (e.g., a spatial index) and devise an efficient algorithmic solution. In section 8, we demonstrate via experimental observations that, for a sufficiently large value of γ, C-MaxRS-Bursty outperforms the event-based processing scheme by an order of magnitude. The basic idea is as follows: we first create a modified slice-based index, *S*_*index*_ for newly occurring γ events (appearing or disappearing objects). Then, we directly add/remove these new events over the existing slice structure *S*_*slice*_ in one iteration, and check the pruning conditions for each slice only once. We describe these ideas in the following section.

### 7.2. Additional Data Structures

**Figure 5:**
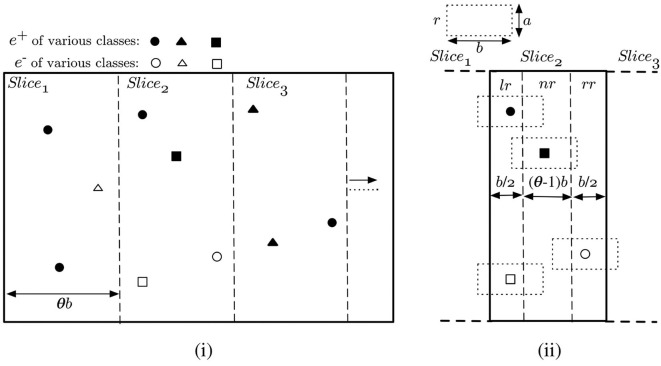
**(i)** Slice indexing over new data and **(ii)** Regions within a slice.

The first step, when handling bursty data updates, is to index the new events based on the locations of their related objects. This allows us later to efficiently retrieve all the new events related to each slice *s*_*i*_ ∈ *S*_*slice*_. Any well-known indexing scheme may be used, e.g., R-tree, Quad-tree, Grid indexing (cf. Ooi et al., 1993; Šidlauskas et al., 2009), etc. To take advantage of the already introduced slice data structure, we propose to use slice-based indexing for the new data. Slice indexing is, basically, a special version of the *p* × *q* grid-indexing—where *q* = 1. Suppose, *S*_*index*_ represents the slice index of new appearing/disappearing objects. Then, we can create *S*_*index*_ as a duplicate of *S*_*slice*_, i.e., width of each slice in *S*_*index*_ is also θ × *b* (where, θ > 1) and |*S*_*index*_| = |*S*_*slice*_| = *s*. An example of the proposed slice indexing is given in Figure 5. Suppose, there are 10 new events occurring at the same time—7 *e*^+^ and 3 *e*^−^, and there are three slices which enclose these event locations. Note that, by event location, we mean the location of the appearing/disappearing object related to the event. In Figure 5i, *Slice*_1_, *Slice*_2_, *Slice*_3_ has, respectively, 3, 4, 3 new events falling within their boundary.

As described in section 5, each of the slices in *S*_*slice*_ track the corresponding rectangles intersecting with them, in addition to the list of maximal slabs, local optimum points and the other attributes. *S*_*index*_, in turn, indexes new events over the slices. An event *e*, corresponding an object *o*_*e*_, is exclusively enclosed by exactly one slice in *S*_*index*_, although the rectangle *r*_*e*_ can overlap with multiple slices. This is illustrated in Figure 5ii. Based on this, we can divide the interior of each slice into three regions:

• **Left-overlapping Region (*lr*):** Rectangles of events in this region overlaps with the left neighboring slice. Width of *lr* is $\frac{b}{2}$. In Figure 5ii, events in *lr* of *Slice*_2_ impact the processing of *Slice*_1_ too.

• **Non-overlapping Region (*nr*):** Rectangles of events in this region are fully enclosed within the slice itself. *nr* is (θ − 1) × *b* wide, i.e., always non-empty as θ > 1.

• **Right-overlapping Region (*rr*):** Rectangles of events in this region overlaps with the right neighboring slice. Width of *rr*, similar to *lr*, is $\frac{b}{2}$. In Figure 5ii, events in *rr* of *Slice*_2_ is also a part of the processing of *Slice*_3_.

Based on the discussion above, each slice *s*_*i*_ ∈ *S*_*index*_ is represented as a 4−tuple (*seq*_*num, E*_*lr*_, *E*_*nr*_, *E*_*rr*_). The role of each attribute is as follows:

- ***seq*_*num***: An integer value assigned to the slice. This encodes the boundary of the slice. For a slice *s*_*i*_, the horizontal extent of *s*_*i*_ is represented by [(*seq*_*num*_*i*_ − 1) × θ*b, seq*_*num*_*i*_ × θ*b*).
- ***E*_*lr*_**: The set of new events in the *lr* region of the slice.
- ***E*_*nr*_**: The set of new events in the *nr* region of the slice.
- ***E*_*rr*_**: The set of new events in the *rr* region of the slice.

Note that, both *S*_*index*_ and *S*_*slice*_ can be merged into one giant slice data structure during implementation. We present them as separate structures here, so that the background motivation and complexity analysis can be clearly demonstrated in the text, i.e., the objective of these two structures are different—*S*_*slice*_ divides the space and overall computation in small slices, while *S*_*index*_ is used only to efficiently index a set of new events.

### 7.3. Processing Bursty Updates

When a collection of new *e*^+^ and *e*^−^ events occur at a time instant, the first step is to initialize and built the slice index *S*_*index*_. Function 3 shows the steps used to build the index from scratch over the new data. In line 1, *S*_*index*_ and *seq*_*num* of its slices are initialized. The other attributes of each slice *s*_*i*_ ∈ *S*_*index*_ is initialized in lines 2–3, i.e., all event lists (based on the region) are set to an empty list. Lines 4–11 iterate though each new events from *E*_*new*_ and set the index attributes accordingly. In line 5, the function retrieves the slice to which *o*_*e*_ belongs, which can be computed in O(1) time. Lines 6–11 find which region *o*_*e*_ is in, and add the corresponding event to the appropriate list. Finally, the newly created index *S*_*index*_ is returned in line 12. The operations from lines 1–3 takes O(s) time, and lines 4–11 takes O(γ) time, where γ is the bursty updates rate. The processing cost of Function 6 is O(γ)+O(s). If we assume γ > *s*, then the overall time-complexity is O(γ).

**Function 3:**
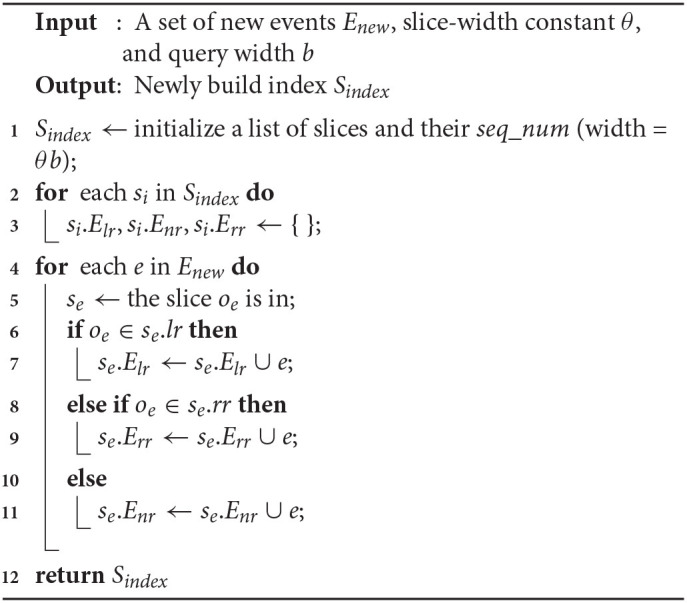
BuildIndex(*E_new_*, *θ*, *b*)

Algorithm 4 shows the steps of our approach for handling a set of new bursty events *E*_*new*_, where |*E*_*new*_| = γ. We combine the pruning ideas of Algorithm 2 and 3, and ensure that *PrepareSlices* and *SliceSearchMR* functions are only called once for these γ new events. In line 1, we use the *BuildIndex* function to prepare the slice index over the new data in O(γ) time. We initialize *S*_*lazy*_, *isPrunable*, and *prev* in lines 2–4. The idea is to traverse the slices from *S*_*slice*_ in one direction, e.g., from left to right. The main idea is that for each slice *s*_*i*_ of *S*_*slice*_, we retrieve the required information of new events from the slice-index *S*_*index*_. The goal is to make sure that we query information of each slice from *S*_*index*_ only once throughout the process. In this regard, we maintain 3 variables—*prev*, *cur*, and *next*—representing the *seq*_*num* − 1, *seq*_*num*, and *seq*_*num* + 1 slices from *S*_*index*_ (new information) any time. Initially, in lines 4–6, *cur* is set to the left-most slice, and *prev* is set to null as there is no slice before that value of *cur*.

Lines 7–29 iterate though each of the slices *s*_*i*_ from *S*_*slice*_ in order (e.g., left to right). At first, information for the (*i* + 1)-th slice index is retrieved into *next*. In line 9, all the related new events of *s*_*i*_ is stored in *E*_*cu*_*r*__*s*_*lice*_, which is the union of new objects in *cur* region, and *prev*.*rr* and *next*.*lr* region (cf. Figure 5ii). In line 10, we check if there are any new events that overlap with the current slice *s*_*i*_—otherwise we move on to the next slice. In lines 12–15, we iterate through the *e*^+^ events of *s*_*i*_—retrieve *r*_*e*_, insert *r*_*e*_ in the *QTree* and add *r*_*e*_ to *s*_*i*_.*R* for each of them. Similarly, lines 16–23 iterate over the *e*^−^ events of *s*_*i*_, although *r*_*e*_ is deleted from *QTree* and *s*_*i*_.*R* in this case. Also, lines 20–22 ensure that *s*_*i*_.*lazy* is set to *true* and *s*_*i*_ is added to *S*_*lazy*_ if *r*_*e*_ overlaps with the local optimum solution. Lines 28 and 29 updates the *prev* and *cur* variables appropriately, and line 30 retrieves the slice *s*_*max*_ containing the global solution. We need to recompute global solution whenever *s*_*max*_.*lazy* = *true* or *isPrunable* = *false* (cf. lines 31 - 33). Finally, the newly computed (or, if pruned, the old) $p_{c}^{*}$ is returned in line 34. In Algorithm 4, each new event is only processed at most 2 times, because θ > 1 and a rectangle *r*_*e*_ can only overlap with at most two slices. Thus, the overall time-complexity of lines 1–30 of Algorithm 4 is O(γ). Also, *PrepareSlices* and *SliceSearchMR* is only processed once for all the new events, instead of worst case γ times via Algorithms 2 and 3. For large values of *N*, the overall processing time of Algorithm 4 is consumed by the execution time of *PrepareSlices* and *SliceSearchMR*.

**Algorithm 4:**
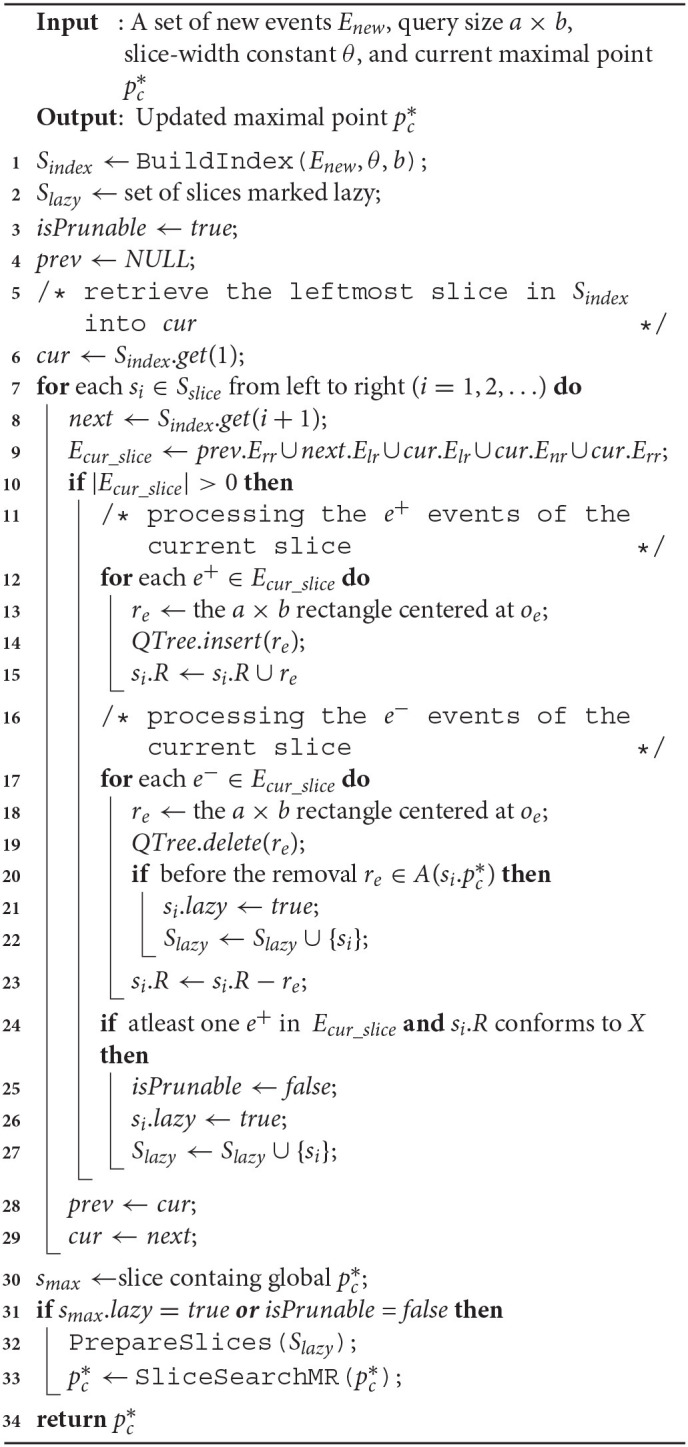
SolveCMaxRSBursty $\left(E_{new},a,b,\theta ,p_{c}^{*}\right)$

### 7.4. Discussion

We presented a slice-based simplified indexing scheme in this section to process a set of bursty events. As slice-indexes are a specialized grid-indexing (see Ooi et al., 1993), they can be implemented both as main-memory or external-memory based. We implemented the proposed slice-indexing in main memory for our experiments. The reason is two- fold. (1) Many recent works such as Kipf et al. (2020) and Šidlauskas et al. (2009) have shown that main-memory indexes are usually necessary to provide high update and build performance—which is paramount in dealing with bursty updates scenarios; and (2) In our experiments, we vary γ from 100 to 100k — which can be stored in-memory. Although, we note that, in extreme scenarios (e.g., Facebook users) where the number of total objects as well as bursty objects surpass the main memory storage capacity of servers, external memory implementations and parallel processing of indexes would be necessary. Many works such as Kamel and Faloutsos (1992) and Kim et al. (2013) presented parallel processing techniques for R-trees and range queries. Kamel and Faloutsos (1992) developed a simple hardware architecture consisting of one processor with several disks to parallelize R-tree processing, where R-tree code is identical to the one for a single-disk R-tree with minimal modifications. Zhong et al. (2012) proposed a novel architecture named VegaGiStore, to enable efficient spatial query processing over big spatial data and concurrent access, via distributed indexing and map-reduce (cf. Dean and Ghemawat, 2008) technique. Recently, SpatialHadoop (Eldawy and Mokbel, 2015) provides a library to perform map-reduce based parallel processing for many spatial operations, including R-tree and grid indexing. We can modify the grid indexing parameters for SpatialHadoop to convert it into a slice-indexing in a straightforward manner. In this way SpatialHadoop can be useful for static scenarios (e.g., Basic C-MaxRS), though the extension to handle dynamic or bursty scenarios is not straight-forward. We note that, Hadoop (Shvachko et al., 2010) and map reduce procedure has a significant overhead, i.e., these will be only be useful if there are a huge number of bursty events, as well as a lot of resources (Hadoop nodes) available.

## 8. Experimental Study

In this section, we evaluate the performance of our algorithms. Since there are no existing solutions, to evaluate our solutions to the C-MaxRS-DU problem, we extended the best known MaxRS solution to cater to C-MaxRS-DU (see section 5.2—i.e., processing the C-MaxRS at each event without any pruning) and used it as a baseline. For bursty streams, we compare the performance of C-MaxRS-Bursty and C-MaxRS-DU, i.e., C-MaxRS-DU becomes the baseline then.

***Dataset***: Due to user privacy concerns and data sharing restrictions, very few (if any) authentic large categorical streaming data (with accurate time information) is publicly available. Thus, we used synthetic datasets in our experiments to simulate spatial data streams. Data points are generated by using both Uniform and Gaussian distributions in a two-dimensional data space of size 1, 000 × 1, 000*m* = *1km*^2^. To simulate the behavior of spatial data streams from these static data points, we use exponential distribution with mean inter-arrival time of 10*s* and mean service time of 10*s*. Initially, we assume that 60% of all data points have already arrived in the system, and use this dataset for static part of evaluation. The remaining 40% of the data points arrive in the system by following exponential distribution as stated earlier. Any data point that is currently in the system, can depart after being served by the system. For experiments related to C-MaxRS-Bursty, we select γ number of events (either in Gaussian or uniform distribution) at any time instant to emulate bursty inputs.

***Parameters***: The list of parameters with their ranges, default values and symbols are shown in Table 1.

**Table 1:** Parameters.

**Parameter name and symbol**	**Possible values**	**Default value**
Object distribution	Uniform, Gaussian	Gaussian
Number of objects, *N*	10k, 20k, 30k, 40k, 50k, 60k, 70k, 80k, 90k, 100k, 200k	50k
Number of POIClass, β	3, 4, 5, 6, 7	5
Min count (per class), μ	1, 2, 3, 4, 5	3
Query area, λ (in m^2^)	100, 225, 400, 625, 900	400
Theta (θ)	1, 2, 3, 4, 5	3
Shape of *R*, *b*:*a*	0.25,0.5,1,2,4	1
Weight, *w*_*i*_	[1, 10]	1
Bursty updates rate, γ	100, 250, 500, 1k, 2.5k, 5k, 10k, 20k,	1k
	30k, 40k, 50k, 60k, 70k, 80k, 90k, 100k	

***Settings***: We have used Python 3.5 programming language to implement our algorithms. All the experiments were conducted in a PC equipped with intel core i5 6500 processor and 16 GB of RAM. We measure the average processing time of monitoring C-MaxRS in various settings. We also compute the performance of Static C-MaxRS computation. In the default settings, the processing time for Static C-MaxRS is 85.86 s. Note that, we exclude the processing time for static C-MaxRS computation in further analysis as this part is similar for both baseline and our approach.

### 8.1. Performance Evaluation: Event-Based Scenario

We now present our detailed observations over different combinations of the parameters for non-bursty scenario (i.e., C-MaxRS-DU).

#### 8.1.1. Varying Number of Objects, *N*

**Figure 6:**
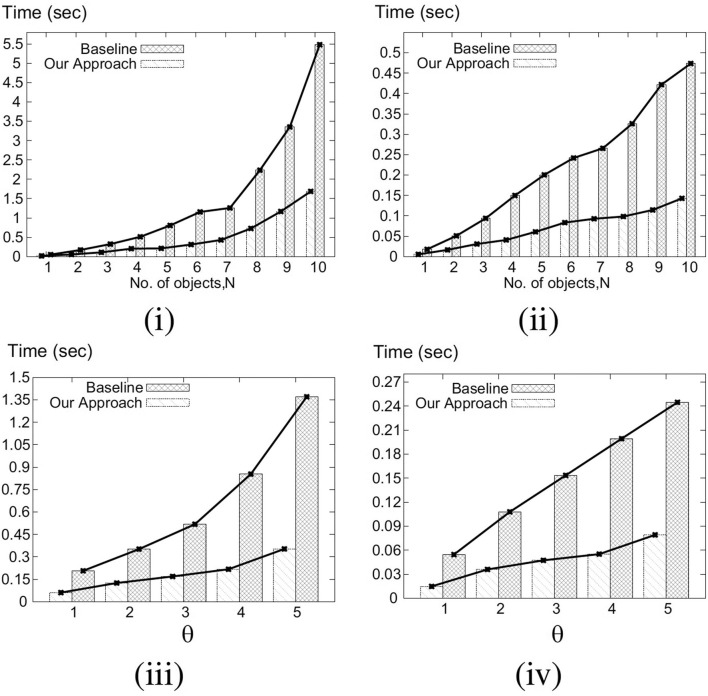
**(i)** Varying *N* for Gaussian. **(ii)** Varying *N* for Uniform. **(iii)** Varying θ for Gaussian. **(iv)** Varying θ for Uniform.

In this set of experiments, we vary the number of objects, *N*, from 10K to 100K (denoted 1–10, respectively, in Figure 6 for brevity, i.e., each label of x-axis needs to be multiplied by 10k), and compare our algorithm with the baseline for different *N* using both Gaussian and Uniform distributions. Figure 6i shows that for Gaussian distribution, the average processing time for our approach (in seconds) increases quadratically (semi-linearly) with the number of objects, whereas the processing time of baseline increases exponentially with the increase of *N*. For Gaussian distribution, on average our approach runs 3.08 times faster than the baseline algorithm. For Uniform distribution, on an average our approach runs 3.23 times faster than the baseline algorithm (Figure 6ii). We also observe that our approach outperforms the baseline in a greater margin for a large number of objects as processing time of our approach increases linearly with *N* for Uniform distribution.

#### 8.1.2. Varying Theta (θ)

Figures 6iii,iv compare the performance of our approach with the baseline by varying theta (θ) for Gaussian and Uniform distributions, respectively. We observe that for both distributions the processing time of baseline algorithm increases at a higher rate than our algorithm, with the increase of θ. Moreover, in all the cases, our approach significantly outperforms the baseline algorithm in the absolute scale/sense. On the average, our approach runs 3.37 and 3.31 times faster than the baseline in Gaussian and Uniform distributions, respectively.

#### 8.1.3. Varying λ - the Area of the Query Rectangle

**Figure 7:**
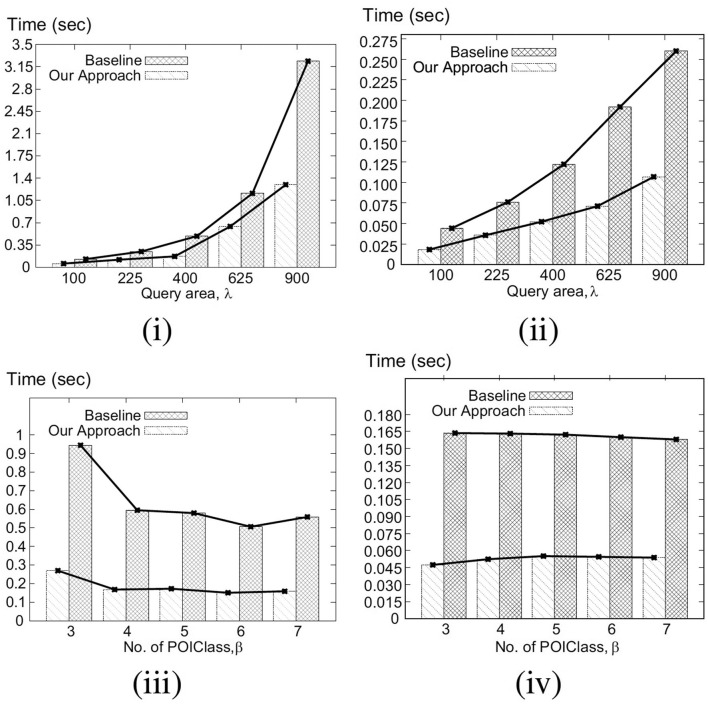
**(i)** Varying λ for Gaussian. **(ii)** Varying λ for Gaussian. **(iii)** Varying β for Gaussian. **(iv)** Varying β for Uniform.

The impact of varying the area of the query rectangle on the average processing times of our approach and baseline algorithm, is shown in Figures 7i,ii. For Gaussian distribution, on an average our approach shows 2.22 times better performance than the baseline approach. Similarly, in Uniform distribution, our approach runs 2.25 times (on average) faster than the baseline. Additionally, note that, as the area of query rectangle increases, corresponding processing time increases as well—due to the possibility of a dual rectangle intersecting with more slices (and other dual rectangles).

#### 8.1.4. Varying POIClass Count, β

The average processing time of our approach and the baseline for varying POIClass Count, β is shown in Figure 7 [Gaussian (iii) and Uniform (iv)]. We observe that the processing time is maximum for the initial case where POIClass Count, β is minimum. Also, we can see that for the both distributions, the processing time decreases with increasing value of β—i.e., handling larger number of classes is faster. On an average our approach runs 3.45 times faster than the baseline algorithm for Gaussian distribution of dataset. In case of Uniform distribution of data, our approach runs 3.06 times faster than the baseline.

#### 8.1.5. Varying Min Class Count, μ

**Figure 8:**
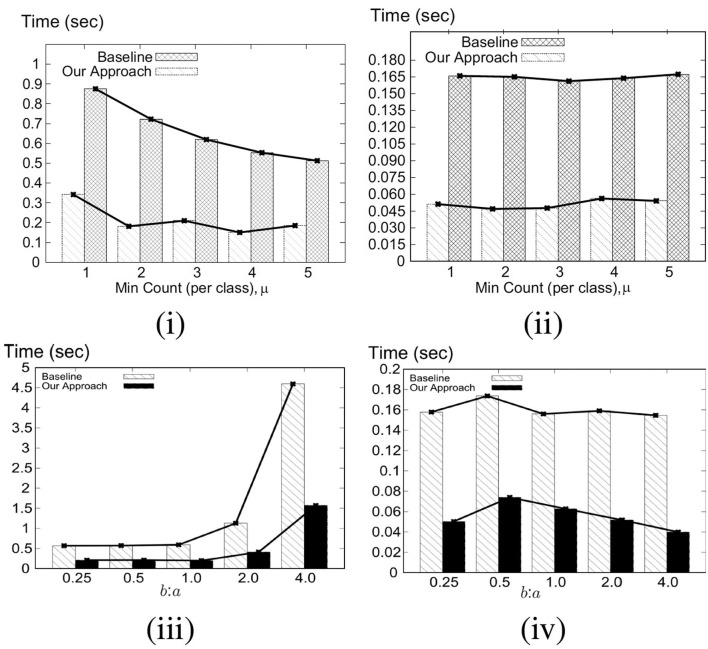
**(i)** Varying μ for Gaussian. **(ii)** Varying μ for Uniform. **(iii)** Varying *b*:*a* for Gaussian. **(iv)** Varying *b*:*a* for Uniform.

Figures 8i,ii show the average processing time of our approach and the baseline by varying Min Class Count, μ. Figures show that for both Gaussian and Uniform distributions, our approach outperforms the baseline significantly. We observe that on an average our approach runs 3.09 and 3.21 times faster than the baseline for Gaussian and Uniform distributions of dataset, respectively. We also note that, the processing time for our approach is largely unaffected by the varying μ values.

#### 8.1.6. Varying Shape of *R*, *b*:*a*

By default, we have used *b*:*a* = 1 in other experiments, i.e., *R* is square-shaped. In this experiment, we investigate whether varying the shape of *R*, i.e., changing the ratio between its width and height, has any effects on the processing time of C-MaxRS-DU. In Figure 8iii for Gaussian distribution, as width (*b*) of *R* is increased, the processing time increases too. This is because, we use θ × *b* as the slice width and as *b* increases, number of slices *s* decreases—reducing the benefits of spatial subdivision. Interestingly, similar trend is not observed in the uniform settings. We note that, in all cases, our approach runs faster than the baseline. In case of Uniform distribution (see Figure 8iv), our approach outruns the baseline approach by 2.99 times on average. In case of Gaussian distribution, our approach outruns the baseline approach by 2.82 times on average.

#### 8.1.7. Comparing Pruning Rules

In this set of experiments, we compare the performance of the different components of our approach. First, we have extended the static C-MaxRS algorithm to handle spatial data streams, which we call the baseline. Then we introduce two pruning rules, one for the appearance event, *e*^+^-Pruning and the other for disappearance event, *e*^−^-Pruning. Finally, we combine both pruning rules to design our approach.

**Figure 9:**
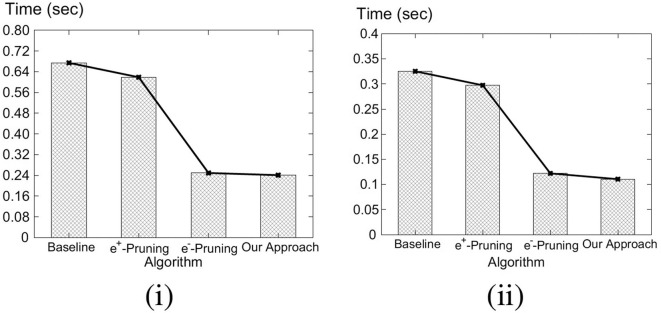
Comparing pruning rules (Unweighted Objects) **(i)** Gaussian and **(ii)** Uniform.

From Figure 9, we can see that *e*^+^-Pruning scheme gives 8.25% performance gain from the baseline algorithm for Gaussian distribution and gives 8.56% performance gain from the baseline algorithm for Uniform distribution of data. The *e*^−^-Pruning scheme provides almost 62.49% performance gain from the baseline for Uniform distribution and 63.01% performance gain from the baseline algorithm for Gaussian distribution.

**Figure 10:**
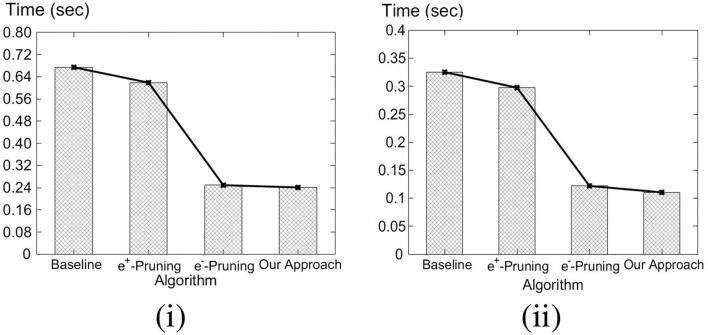
Comparing pruning rules (Weighted Objects) **(i)** Gaussian and **(ii)** Uniform.

We also perform this experiment using weighted objects, where each object is assigned with a random weight. We vary the weights of the objects from 1 to 10. In Figure 10, we see similar trends among the evaluated algorithms. Also, we note that, the processing time is faster for the weighted experiments. It is because, due to the variance in the weights of objects, more events can be pruned easily. This experiment also validates our analysis in section 6.

### 8.2. Performance Evaluation: Bursty Streaming Updates

We now present our detailed observations over different combinations of the parameters for bursty updates (i.e., C-MaxRS-Bursty vs. C-MaxRS-DU).

#### 8.2.1. C-MaxRS-Bursty vs. C-MaxRS-DU

**Figure 11:**
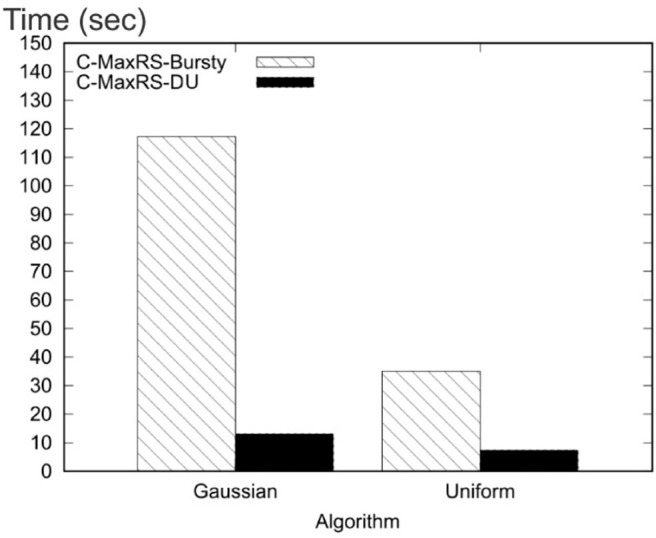
Comparing *C*-MaxRS-DU and *C*-MaxRS-Bursty for default settings.

We present the performance comparison (over both distribution of data) for C-MaxRS-DU and C-MaxRS-Bursty in Figure 11 in default settings (i.e., γ = 1000). We can see that C-MaxRS-Bursty is way more efficient than C-MaxRS-DU in handling bursty streams in both distributions, i.e., C-MaxRS-Bursty is almost 5 and 10 times faster than C-MaxRS-DU in the default settings for uniform and Gaussian distribution of data, respectively.

#### 8.2.2. Varying γ

**Figure 12:**
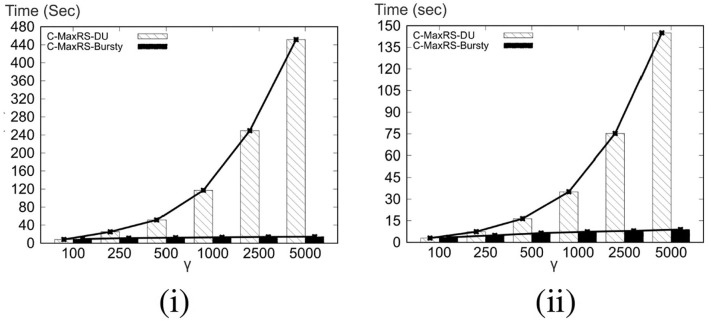
Varying γ **(i)** Gaussian and **(ii)** Uniform.

We change the value of the bursty streaming rate, γ, from 100 to 5,000. Figure 12 shows the total processing time (in seconds) of γ events together. In Figure 12i (uniform distribution), initially when γ = 100, C-MaxRS-DU (2.99 s) performs better than C-MaxRS-Bursty (3.51s). But, for γ = 250, C-MaxRS-Bursty performs faster, i.e., 7.4 vs. 4.88 s. Thus, for this setting, there is a value of γ in-between 100 and 250, after which C-MaxRS-Bursty starts out-performing C-MaxRS-DU. This aligns with our intuition that for cases where γ is not too high, C-MaxRS-DU gives us the optimal performance, whereas, C-MaxRS-Bursty is more efficient as γ increases.

In Figure 12, as the value of γ increases, the processing time for C-MaxRS-DU increases exponentially, but the increase in C-MaxRS-Bursty is linear. C-MaxRS-Bursty outperforms C-MaxRS-DU by 5.89 times on average for uniform distribution of data, and by 10.94 times in case of Gaussian distribution of data. This experiment shows the effectiveness of C-MaxRS-Bursty for high streaming data.

#### 8.2.3. Varying *N*

**Figure 13:**
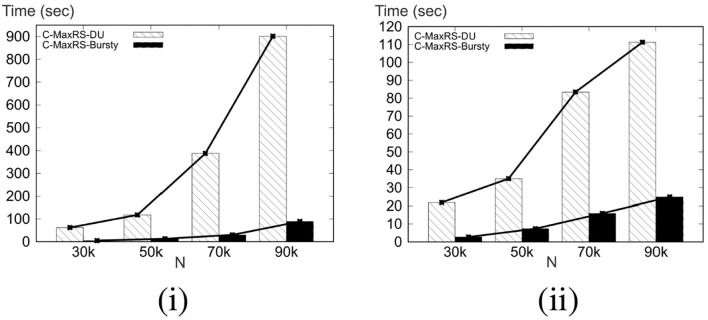
Varying *N* **(i)** Gaussian and **(ii)** Uniform.

Subsequently, we vary the value of *N*, i.e., number of objects, and preset the results in Figure 13. Processing times of both the algorithms increase with the increasing cardinality, although, we note that the increase in C-MaxRS-Bursty is much slower. C-MaxRS-Bursty outperforms C-MaxRS-DU by 5.60 times on average for uniform distribution of data. For Gaussian distribution, C-MaxRS-Bursty outperforms C-MaxRS-DU by 11.34 times on average.

#### 8.2.4. Scalability of C-MaxRS-Bursty

**Figure 14:**
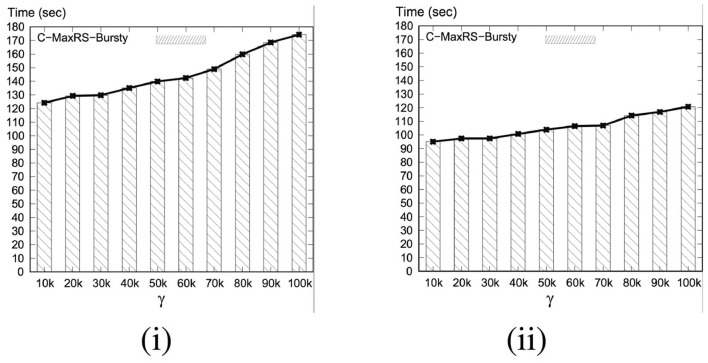
Varying γ in larger scale **(i)** Gaussian and **(ii)** Uniform.

In the final experiment, we show the effect of larger γ values on C-MaxRS-Bursty in Figure 14. We also use a larger value of *N* for this experiment–i.e., the value of γ is varied from 10, 000 to 100, 000, and the total number of objects *N* is set to 200, 000. We omit the performance of C-MaxRS-DU for this experiment as the processing time for large γ values is exponentially high (to avoid skewing the graph). We can see that, the results in Figure 14 illustrate similar trend as Figure 12, even though we used significantly larger values of γ and *N*. For both distributions, processing time increases only slightly as the value of γ increases. For example, in Figure 14i, for a 10 times increase of γ value (from 10 k to 100k), the processing time only increases by 1.4 times (from 124.2 to 174.3 s). Same is true for uniform distribution (cf. Figure 14i), where this increase is even less (1.27 times, i.e., from 95.1 to 124.8 s). We also note that, the bulk of the processing time of C-MaxRS-Bursty is consumed by lines 32–33 of Algorithm 4–i.e., executing the function *PrepareSlices* and *SliceSearchMR*. These results demonstrate the scalability of C-MaxRS-Bursty – where it is ensured that recomputation (i.e., lines 32–33) is performed only once (in worst case) instead of γ times.

## 9. Conclusions and Future Work

In this paper, we have proposed a new variant of MaxRS query, namely *Conditional Maximizing Range-Sum* (C-MaxRS) query in spatial data streaming updates for both non-weighted and weighted objects. Initially, we simply adapted the traditional MaxRS settings to incorporate conditional constraints of different class of objects. However, to handle data updates (i.e., appearance and disappearance of objects) with class-awareness, we needed additional spatial data structures, quadtree and a variant of self-balancing binary tree (e.g., we used AVL-tree), which enabled our algorithm to efficiently compute the changes in the result for different partitions (or slices) of the dataspace. To further improve the overall time-efficiency, we developed two pruning rules: one to handle the appearance of an object and the other to handle disappearance of an object while updating C-MaxRS results. Additionally, to accommodate a different kind of applications settings where a bursty stream of data updates occur in a short time interval, we have proposed a novel technique, C-MaxRS-Bursty to efficiently compute the C-MaxRS results via bulk updates handling. We considered a large parameters space and conducted extensive set of experiments. In sequential spatial data stream scenario, our approach, C-MaxRS-DU yields three to four times improvements (on average) in terms of processing time, when compared to the baseline algorithm. We have also observed that in a bursty scenario, our approach C-MaxRS-Bursty outperforms our one-at-a-time approach, C-MaxRS-DU, by 5–10 times.

There are several immediate extensions to our work. Firstly, we would like to investigate the trade-offs arising when there is a constraint between the time-instant of a particular update and the update of the answer. This, in some sense, may require a new approach where the bulk update algorithms and data structures proposed in this work will need to be adapted to handle dynamic invocations (e.g., when the buffer of new data reaches certain capacity). Complementary to this, we plan to investigate the C-MaxRS in more traditional streaming settings—i.e., when there is a constraint on the memory and the arrival rate is explicitly taken in consideration. In such cases, relying on data sketches may be inevitable (similar to Cormode, 2017). Lastly, we are investigating the variations of C-MaxRS where different kinds of mobility may need to be incorporated—for both the users (cf. Hussain et al., 2017a) and the query rectangle (e.g., in the Loon Project settings), as well as the mutual dependencies of both.

## Data Availability Statement

The datasets and the code used in the experiments are publicly available at: https://users.cs.northwestern.edu/~mmh683/project-works/Conditional-MaxRS-Streams/.

## Author Contributions

All authors listed have made a substantial, direct and intellectual contribution to the work, and approved it for publication.

## Conflict of Interest

The authors declare that the research was conducted in the absence of any commercial or financial relationships that could be construed as a potential conflict of interest.

## Footnotes

^1^cf. https://www.census.gov/topics/income-poverty/income.html.

^2^The Loon project (formerly Google X goo, 2016) aims at providing internet access to remote/rural areas via a collection of high-altitude balloons providing wireless networks with up to 4G LTE speeds.

^3^More specifically, Feng et al. (2016) was considering submodular monotonic functions as aggregates.
